# Analysis of aquaporins from the euryhaline barnacle *Balanus improvisus* reveals differential expression in response to changes in salinity

**DOI:** 10.1371/journal.pone.0181192

**Published:** 2017-07-17

**Authors:** Ulrika Lind, Michael Järvå, Magnus Alm Rosenblad, Piero Pingitore, Emil Karlsson, Anna-Lisa Wrange, Emelie Kamdal, Kristina Sundell, Carl André, Per R. Jonsson, Jon Havenhand, Leif A. Eriksson, Kristina Hedfalk, Anders Blomberg

**Affiliations:** 1 Department of Marine Sciences, Lundberg laboratory, University of Gothenburg, Gothenburg, Sweden; 2 Department of Chemistry and Molecular Biology, Lundberg laboratory, University of Gothenburg, Gothenburg, Sweden; 3 Department of Marine Sciences, National Infrastructure of Bioinformatics (NBIS), Lundberg laboratory, University of Gothenburg, Gothenburg, Sweden; 4 Department of Molecular and Clinical Medicine, Wallenberg Laboratory, Sahlgrenska Academy, University of Gothenburg, Gothenburg, Sweden; 5 RISE Research Institute of Sweden, Section for Chemistry and Materials, Borås, Sweden; 6 Department of Biological and Environmental Sciences, University of Gothenburg, Gothenburg, Sweden; 7 Department of Marine Sciences-Tjärnö, University of Gothenburg, Strömstad, Sweden; Universita degli Studi di Bari Aldo Moro, ITALY

## Abstract

Barnacles are sessile macro-invertebrates, found along rocky shores in coastal areas worldwide. The euryhaline bay barnacle *Balanus improvisus* (Darwin, 1854) (= *Amphibalanus improvisus*) can tolerate a wide range of salinities, but the molecular mechanisms underlying the osmoregulatory capacity of this truly brackish species are not well understood. Aquaporins are pore-forming integral membrane proteins that facilitate transport of water, small solutes and ions through cellular membranes, and that have been shown to be important for osmoregulation in many organisms. The knowledge of the function of aquaporins in crustaceans is, however, limited and nothing is known about them in barnacles. We here present the repertoire of aquaporins from a thecostracan crustacean, the barnacle *B*. *improvisus*, based on genome and transcriptome sequencing. Our analyses reveal that *B*. *improvisus* contains eight genes for aquaporins. Phylogenetic analysis showed that they represented members of the classical water aquaporins (Aqp1, Aqp2), the aquaglyceroporins (Glp1, Glp2), the unorthodox aquaporin (Aqp12) and the arthropod-specific big brain aquaporin (Bib). Interestingly, we also found two big brain-like proteins (BibL1 and BibL2) constituting a new group of aquaporins not yet described in arthropods. In addition, we found that the two water-specific aquaporins were expressed as C-terminal splice variants. Heterologous expression of some of the aquaporins followed by functional characterization showed that Aqp1 transported water and Glp2 water and glycerol, agreeing with the predictions of substrate specificity based on 3D modeling and phylogeny. To investigate a possible role for the *B*. *improvisus* aquaporins in osmoregulation, mRNA expression changes in adult barnacles were analysed after long-term acclimation to different salinities. The most pronounced expression difference was seen for AQP1 with a substantial (>100-fold) decrease in the mantle tissue in low salinity (3 PSU) compared to high salinity (33 PSU). Our study provides a base for future mechanistic studies on the role of aquaporins in osmoregulation.

## Introduction

Aquaporins (AQPs) are pore-forming integral membrane proteins that mainly facilitate transport of water and small solutes through cellular membranes. Aquaporins arose early in evolution and exist across all phyla [1, 2], indicating their fundamental importance in cell function. The number of aquaporins varies widely between species; there are two in the bacterium *Escherichia coli*, four in the yeast *Saccharomyces cerevisiae*, eight in the fruit fly *Drosophila melanogaster*, thirteen in humans and even higher numbers in plants with seventy-one paralogs in cotton [2–4].

The superfamily of eukaryotic aquaporins is phylogenetically divided into four main subfamilies (referred to as "grades" [2]) based on their sequences: The classical aquaporins that mainly transport water, the aquaglyceroporins that transport glycerol and other small molecules, the aquaammoniaporins that transport ammonia and other small molecules, and the unorthodox aquaporins that are, at this stage, not well functionally characterized [2]. In addition to the water-transporting aquaporins, the classical aquaporins includes two arthropod specific subfamilies, big brain (BIB) and Eglp, for which water is not the main substrate [2]. BIB does not transport water [5] but has been reported to take part in ion conductance and cell-adhesion [6, 7]. Ion transport and cell-adhesion properties have also been demonstrated for some of the vertebrate water-transporting aquaporins [8, 9]. Eglps are glycerol transporting aquaporins that are present in holometabolous insects where they evolved from water transporting aquaporins and replaced typical aquaglyceroporins [10]. Sequence-function relationship between the various classes is thus in some cases quite complex. This evolutionary complexity makes it essential to experimentally determine the functionality of newly identified aquaporins, especially for groups of organisms that are not well studied.

Aquaporins consist of six transmembrane-spanning helices connected by five loops and have cytoplasmic N- and C-termini. One cytoplasmic loop and one extracellular loop are partly embedded in the plasma membrane as half-helices and contain the two highly conserved asparagine-proline-alanine (NPA) motifs. The NPA motifs are localized in a central constriction of the aquaporin channel and is the key selectivity filter that allows the passage of water while at the same time excludes protons and cations [2]. Substrate specificity is partially determined by the Ar/R (aromatic/arginine) constriction site that is located closer to the entrance of the pore than the NPA sites, and is the narrowest part of the channel. The Ar/R constriction site usually consists of four amino acids including an aromatic amino acid and an arginine [11, 12].

It is well established that aquaporins play very diverse physiological roles in organisms. The aquaporins in humans have been shown to be important for water homeostasis and osmoregulation in several organs, such as kidney, brain, adipose tissue and skin [13]. AQP2 for example, plays a critical role in water resorption in the kidney and mutations in the *AQP2* gene cause kidney dysfunction and development of the disease nephrogenic diabetes insipidus [14]. In mice, *AQP7* null mutations lead to obesity [15]. In the malaria parasite *Plasmodium berghei* deletion of the sole aquaporin gene negatively affects growth of the parasite and results in reduced pathology [16]. Deletion of the classical aquaporins in yeast has consequences for intra-cellular ice-formation and leads to higher mortality from freeze-thawing regimes [17]. QTL mapping and RNAi established that aquaporin affects the fecundity of female *D*. *melanogaster* [18]. Thus, physiological importance of aquaporins has been shown for most branches of the tree of life.

In crustaceans, a possible role of aquaporins in osmoregulation has been indicated by their transcriptional regulation upon salinity stress in the crabs *Portunus trituberculatus* and *Callinectes sapidus* and in the shrimp *Litopenaeus vannamei* [19–21]. The repertoire and function of the aquaporins in the marine blood-feeding copepod *Lepeophtheirus salmonis* (salmon louse) have recently been presented [22]. This crustacean encodes seven aquaporins representing all major aquaporin families except the AQP8-like aquaammoniaporins, with two of the three aquaglyceroporins being expressed as splice variants. Analysis of the permeation properties of the salmon louse aquaporins showed their sequence-predicted functions; e.g. the classical aquaporins mainly transported water, and the aquaglyceroporins were shown to transport glycerol, water and urea [22]. The complete aquaporin repertoire is available also for the genome sequenced branchiopod *Daphnia pulex* (NCBI NR data base).

Barnacles (subphylum Crustacea, subclass Thecostraca, infraclass Cirripedia) are sessile macro-invertebrates, commonly found along rocky shores in coastal areas worldwide. Barnacles produce free-swimming larvae that go through several larval stages, of which the last is the cyprid that attaches to hard substrates and develops into a sessile adult. The euryhaline bay barnacle *Balanus improvisus* (Darwin, 1854) (= *Amphibalanus improvisus*) can tolerate a wide range of salinities; both adults and larvae can thrive from close to freshwater (3 PSU) up to fully marine conditions (35 PSU) [23–25] and survive fluctuating salinities [26, 27]. Several studies have reported that *B*. *improvisus* is a true “brackish” species with optimal performance at intermediate salinities [28, 29], and beyond the Danish Straits and the Belt Sea it is the only barnacle in the brackish environment of the Baltic Sea [30]. Although much is known about the ecology of *B*. *improvisus*, the physiological/molecular mechanisms responsible for its exceptional tolerance to a wide range of salinities are not well understood. We have previously cloned and characterized the primary transporter Na^+^/K^+^-ATPase and examined its possible involvement in osmoregulation in *B*. *improvisus*. This important ion transporter is encoded from a number of paralogous genes where some are expressed as salinity-regulated splice variants [31]. In this paper, we present the whole repertoire of aquaporins from *B*. *improvisus* based on genome and transcriptome sequencing. We functionally characterize some of the AQP paralogs by investigating their ability to transport water and glycerol, model their 3D structure *in silico*, and investigate aquaporin expression in relation to salinity. Our analysis provides the first characterization of aquaporins within the subclass Thecostraca revealing the presence of a new aquaporin group (BIB-like) as well as differential expression of water transporting AQPs during osmoregulation.

## Results

### Identification and phylogenetic classification of barnacle aquaporins

To identify barnacle aquaporins, human and *Drosophila* aquaporin protein sequences were compared to genome and transcriptome assemblies of *B*. *improvisus* (assemblies to be published separately; manuscript in preparation) using BLAST. Eight different genes encoding aquaporins were found, and the mRNA sequences of the open reading frames were confirmed by PCR or by independent transcriptome data. Due to high genetic diversity with 3–4% pair-wise nucleotide variation between alleles, different alleles were scored for each gene. However, the allelic variation between individuals will be examined in more detail in later population studies.

**Fig 1:**
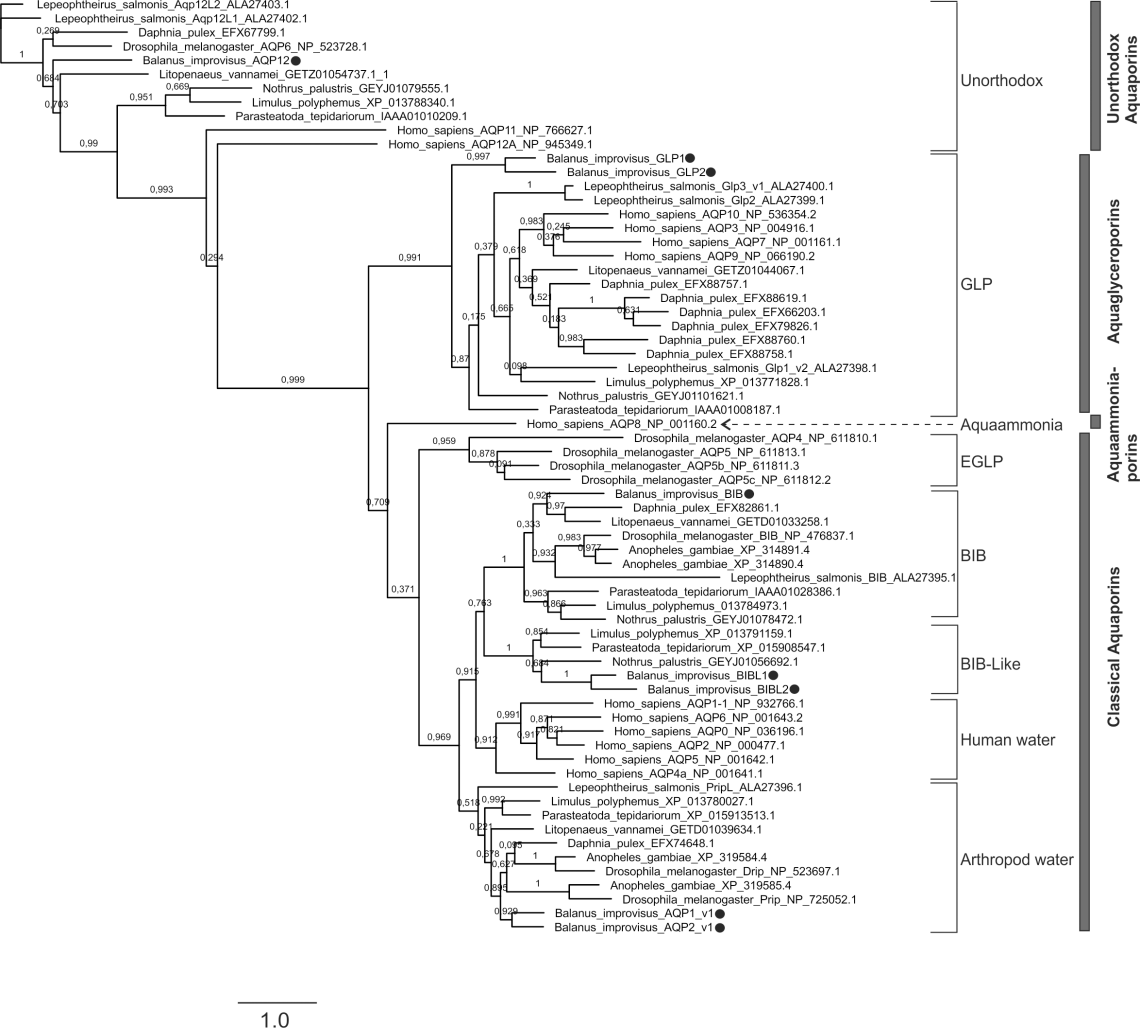
Initial phylogenetic classification of the aquaporins in *B*. *improvisus*. A phylogenetic tree was constructed using aquaporin sequences from *B*. *improvisus* and other arthropods. In addition, the human aquaporins were included for reference. The analysis was done using the program PhyML 3.0 at the Phylogeny.fr website, creating an unrooted tree. The four main subfamilies according to Stavang et al [22] are indicated to the right and different aquaporin subgroups to the left. Aqp8-type aquaammoniaporins are abbreviated to aquaammoniaporins. The *B*. *improvisus* aquaporins are marked with a dot. The numbers on the branches are aLRT SH-like support-values. The scalebar shows substitutions per site.

Initial phylogenetic classification indicated that two of the eight *B*. *improvisus* aquaporins belong to the water specific AQPs within the classical aquaporin subfamily (named Aqp1 and Aqp2), two belong to the aquaglyceroporins (named Glp1 and Glp2), one to the unorthodox aquaporins (named Aqp12) and one belong to the arthropod-specific aquaporin class called big brain within the classical aquaporin subfamily (named Bib, Fig 1). The last two of the *B*. *improvisus* aquaporins are in a clade most closely related to big brain and were named Bib-like1 (BibL1) and Bib-like2 (BibL2) (Fig 1). An extended phylogenetic analysis using Bayesian inference, including 264 water- and big brain-related aquaporins from arthropods, also showed that the BibL1 and BibL2 aquaporins are most closely related to big brain (S1A and S1B Fig). The support value for the big brain and big brain-like clades was high (support 100) and they were distinct from the other classical aquaporins (support 100); these phylogenetic branches were confirmed performing a maximum likelihood analysis on the same set of sequences (support 97–100, data not shown).

In hexapods two different classes of aquaglyceroporins have been identified, classical GLPs and the independently evolved eGLPs [10]. In holometabolous insects, the ancestral branch of aquaglyceroporins was replaced by eGLPs during evolution, whereas some other hexapods have both GLPs and eGLPs. In contrast, crustaceans have been reported to only contain classical GLPs [10, 22]. The Glp1 and Glp2 from *B*. *improvisus* clearly belong to the classical GLP clade, with the two paralogs found in a separate branch indicating a rather recent duplication (Fig 1). Similar to salmon louse [22], we did not find any indications of the presence of an Aqp8-type aquaammoniaporin in *B*. *improvisus*.

### Barnacle AQP exon/intron gene structure

**Fig 2:**
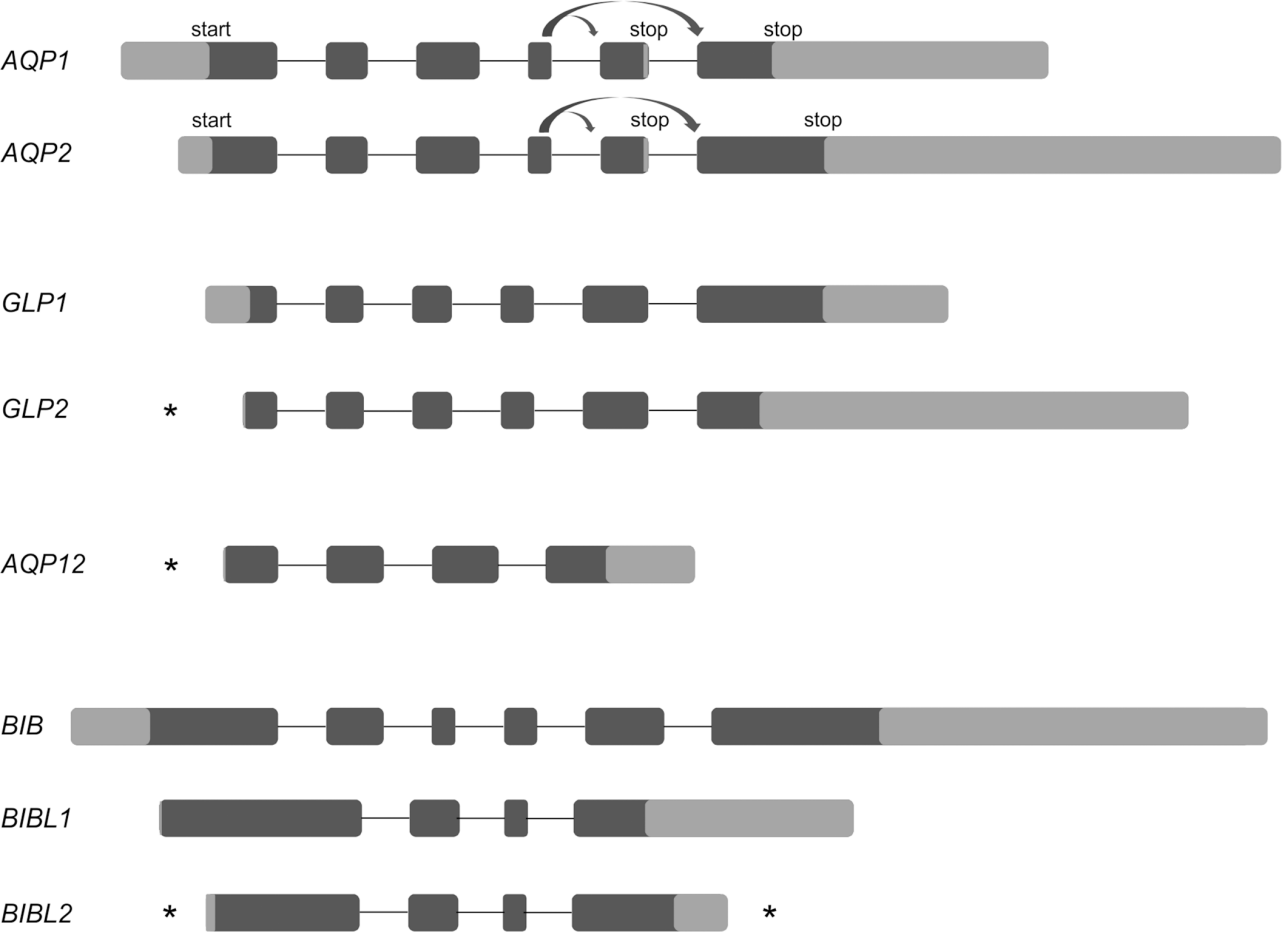
Gene structure of the eight aquaporin genes in *B*. *improvisus*. The *B*. *improvisus* aquaporin genes contain 4–6 exons. *AQP1* and *AQP2* are expressed as two alternative splice variants where exon 5 is excluded in one of the isoforms (indicated by bent arrows). Coding parts of exons are indicated in black, 5’ and 3’ UTR regions in grey and introns by thin lines. Exons, but not introns, are displayed to scale. For indication of intron lengths see S2 Fig. The minimum lengths of the UTRs being part of the first or last coding exon are shown. An asterisk indicates that the UTR continues in an upstream or downstream non-coding exon not shown in the figure. Genomic data, cDNA clones and RNA-seq data were used to define the gene structure. In the case of the BIBL1 and BIBL2 mRNA, the sequence of the complete 3´end was determined by RACE.

In order to better understand the evolution of the AQP genes in *B*. *improvisus*, we analyzed the intron/exon arrangement of each gene. Gene exon/intron structures are typically highly conserved among homologous genes in recently duplicated genes [32] and the analysis of these structures thus provides clues to the evolutionary history of gene families [33]. Mapping of AQP mRNA sequences to the genome contigs revealed that the coding regions of *B*. *improvisus* aquaporins consist of 4 or 6 exons (Fig 2); *AQP1*, *AQP2*, *GLP1*, *GLP2* and *BIB* consist of 6 exons, and *BIBL1*, *BIBL2* and *AQP12* contain 4 exons. Exon length covered the range 50–500 bp, with the exception of the first and last coding exons that usually are longer due to addition of untranslated regions (UTRs) before and after the translation start and stop. The longest exon was exon VI of *AQP2* that is at least 1900 bp. The length of the introns is highly variable spanning from 200 bp to almost 6 kbp (S2 Fig). We noted that the intron/exon borders were identical both for amino acid position and intron phase for each of the paralogous pairs *AQP1* and *AQP2*, *GLP1* and *GLP2*, and *BIBL1* and *BIBL2* indicating rather recent duplications.

A comparison of the exon/intron borders of the *B*. *improvisus* aquaporins with the corresponding exon/intron borders of the aquaporins in salmon louse and *Daphnia* showed similar exon/intron architecture in several cases. This would indicate a common evolutionary history in the different species for some of the aquaporin classes with no drastic gene rearrangements after the ancient evolutionary splits. For example, *AQP12* has four exons in both *B*. *improvisus* and salmon louse and show an identical exon/intron architecture in the two species. In the case of the classical water-transporting aquaporins, both the *B*. *improvisus AQP1* and *AQP2* have six coding exons, where the first five exons have identical exon/intron architecture as the five exons of the water-transporting AQP in *D*. *pulex*. *PripL* in the salmon louse has four coding exons with identical exon/intron borders as *AQP1* and *AQP2*, except that the first exon is merged and corresponds to exon 1 and exon 2 in the *B*. *improvisus AQP1* and *AQP2*.

For the aquaglyceroporins, two of the six *Daphnia* GLPs shared exactly the same exon/intron borders with the *Balanus* GLPs and the other four are largely similar to those of the *Balanus* GLPs, but displaying merged or split exons. The three GLPs in salmon louse on the other hand showed a complete different exon/intron structure with fewer exons than the GLPs in salmon louse and *B*. *improvisus* and with exon/intron borders that did not overlap at all. Interestingly, the coding sequence for the big brain aquaporin in *B*. *improvisus* is composed of several exons while the salmon louse homolog consists of only one single exon. Since other arthropods also have a big brain gene structure composed of several exons, the single exon gene in the salmon louse big brain seems to be the exception and was suggested to be a result of a replacement event that occurred via reverse transcription and exclusion of an older exon/intron split gene [22].

**Fig 3:**
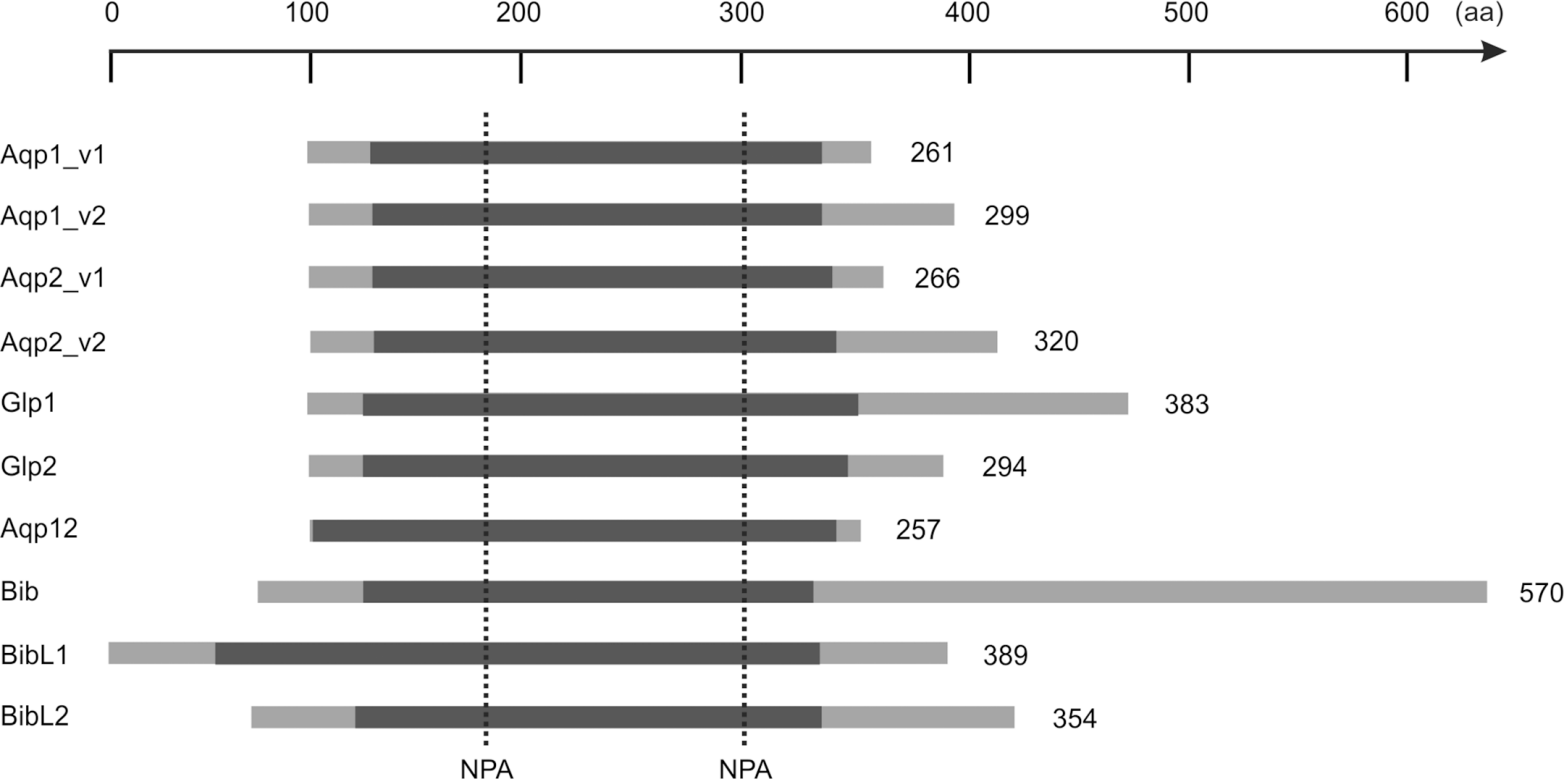
Schematic overview of the *B*. *improvisus* aquaporins. The length [number of amino acids (aa)] and main features of the aquaporins in *B*. *improvisus* are displayed. Included are also the splice forms for Aqp1 and Aqp2. The region spanning from transmembrane 1 to transmembrane 6 (see Fig 4), is indicated in dark grey and the N- and C-termini in light grey. The sequences are roughly aligned according to the position of the NPA sites. The total number of amino acid residues for each aquaporin is indicated to the right.

In the case of AQP1 and AQP2 we found two different variants for each aquaporin in the RNA seq data from one single individual, differing only in the C-termini. The two variants are clearly the result of alternative splicing since we find no evidence for the existence of more than one AQP1 gene and one AQP2 gene in the genome sequence. The same type of splicing occurs for both genes, either including or excluding the second last exon (exon V). The shorter C-terminal splice isoforms, Aqp1_v1 and Aqp2_v1, include exon V that contains a stop codon and exon VI is thus in this case not translated. Exon VI has an alternative stop codon used when exon V is excluded, producing the longer isoforms Aqp1_v2 and Aqp2_v2 (Figs 2 and 3).

### Conserved motifs in *B*. *improvisus* AQPs

The members of the aquaporin family in *B*. *improvisus* range in size between 257 and 570 amino acids (Fig 3). Their N-terminal regions vary only somewhat in length, while the length of the C-terminal regions varies greatly. The Bib C-terminus consists of about 300 residues, which is in line with the characteristic very long C-termini present in the *Drosophila* [5], salmon louse [22], and *Daphnia* (EFX82861.1) big brain aquaporins. The two Bib-like proteins (BibL1 and BibL2) in *B*. *improvisus*, however, do not contain the correspondingly long C-terminus (Fig 3). In *Drosophila* BIB, and also in human AQP1, tyrosine phosphorylation of the carboxyl terminal domain has been shown to modulate aquaporin ion channel activity [6, 34]. In the C-terminus of *Drosophila* big brain there are seven tyrosines that are to a large part conserved among insect BIBs, which could be potential tyrosine phosphorylation sites [5]. All of those are also conserved in the C-terminus of the *B*. *improvisus* Bib. Interestingly, there is a 26 residues motif including three of these tyrosines (Tyr343, Tyr350 and Tyr368 in *B*.*improvisus*), which is almost 70% identical between the two species (S3 Fig), potentially indicating functional importance across long evolutionary distance.

**Fig 4:**
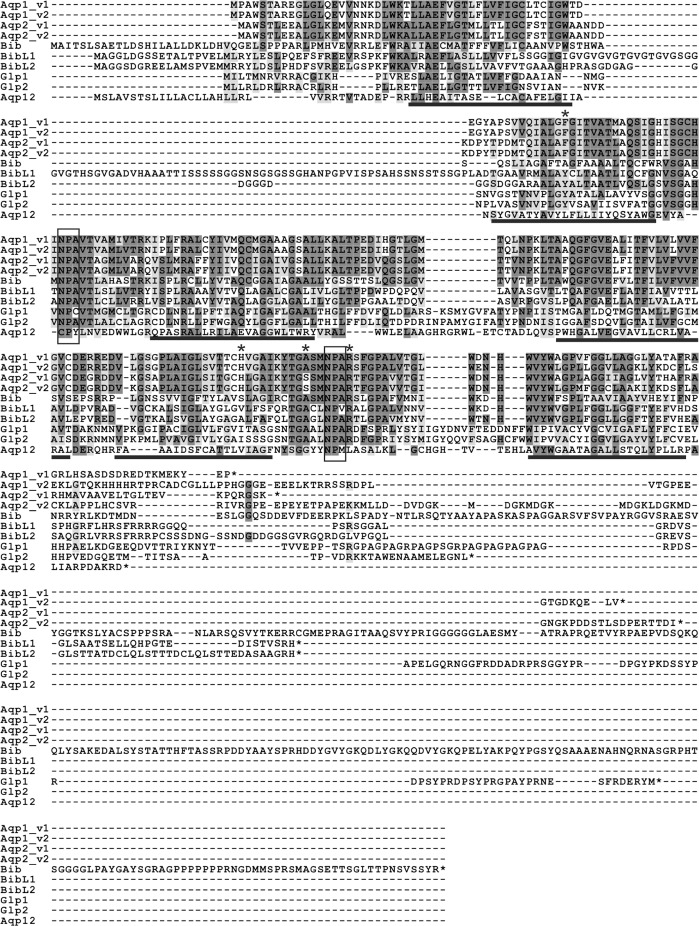
Protein alignment of the aquaporins in *B*. *improvisus*. All aquaporins from *B*. *improvisus*, including splice variants, are aligned. Dark grey indicates identical amino acids and light grey functionally similar amino acids. The positions of the transmembrane spanning regions, based on TMHMM predictions of Aqp1_v1, are indicated with a black line under the sequences. The two NPA motifs (or the variants thereof) are indicated with boxes, and the four amino acids in the constriction site (see Fig 5) are indicated with an asterisk. Some sequence differences are apparent between Aqp1_v1 and Aqp1_v2 even outside the C-terminal portion corresponding to exon 5 and 6. This is because the two sequences are obtained from different individuals.

Sequence analysis using the transmembrane helix prediction programs TMHMM [35, 36] and TMpred indicated that all *B*. *improvisus* aquaporins contain six transmembrane helices (TMHMM predictions for all aquaporins are displayed in S4A Fig). However, in the case of Aqp12 the probability scores for some of the helices were weak which is in line with AQP12 proteins from other species (S4B Fig). In addition, sequence analysis of the *B*. *improvisus* aquaporins showed that they all contain the two conserved NPA motifs (Fig 4) believed to be involved in proton exclusion [2]. However, for some of the *B*. *improvisus* AQPs the NPA motifs deviate from the canonical NPA sequence; the unorthodox aquaporin Aqp12 have instead CPY and NPM in the first and second motif, respectively, the aquaglyceroporin Glp1 has NPC in its first motif and BibL1 has NPV in its second motif (Fig 4). The variations seen in the NPA motifs in the *Balanus* AQPs are not unusual in comparison to AQPs from other species. Large deviations from the canonical NPA sequence are commonly found in AQP12 proteins, and NPC and NPV substitutions are found in aquaporins that have been shown to be functional in *in vitro* assays [37, 38]. NPC is also commonly found in AQP11 orthologues of different vertebrate species, however, conflicting results regarding its proposed water-transporting function have been reported [39, 40].

**Fig 5:**
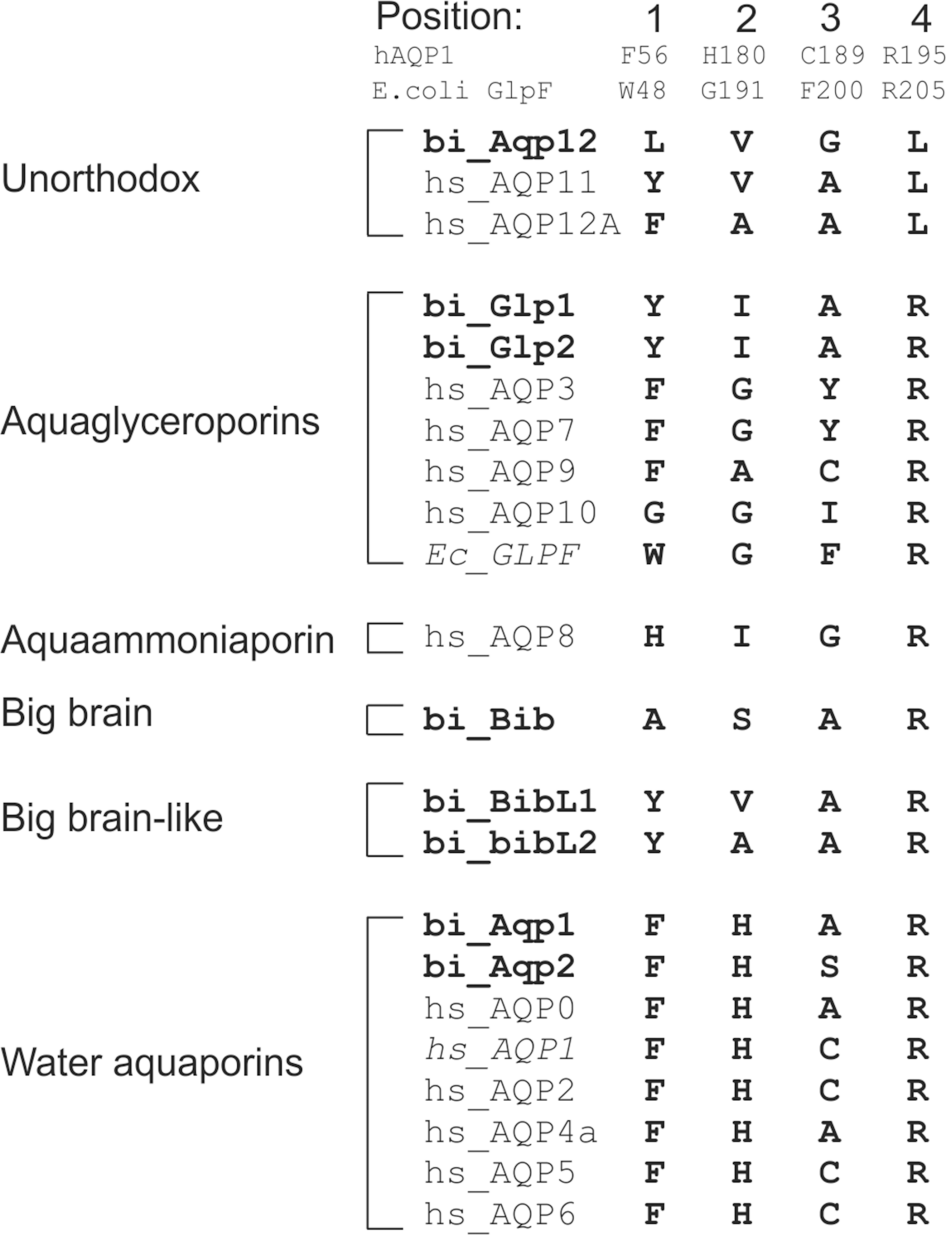
Amino acids in the Ar/R constriction site of aquaporins in *B*. *improvisus*. The four amino acids in the Ar/R constriction motif in the *Balanus* aquaporins were identified based on protein alignments with the human water aquaporin AQP1 and the *E*. *coli* aquaglyceroporin Glpf, which have well characterized constriction regions. All human aquaporins are included for comparison. Positions and residues for human AQP1 and *E*. *coli* Glpf are indicated at the top. The names of the *B*. *improvisus* aquaporins are shown in bold.

Positions for the four amino acids in the Ar/R constriction motif in the *Balanus* aquaporins were identified based on protein alignments with the human water aquaporin AQP1 and the *E*. *coli* aquaglyceroporin Glpf (Fig 5), both of which have previously been crystallized and their 3D structure determined [11, 12]. In the most highly conserved fourth position in the constriction site, all *B*. *improvisus* aquaporins, except Aqp12, have an arginine. This arginine has been suggested to be involved in proton exclusion together with the NPA motifs [41]. Similar to most aquaglyceroporins, the *B*.*improvisus* Glp1 and Glp2 have an aspartate following the arginine in the constriction region (Fig 4). In the first position in the Ar/R constriction motif, all *B*. *improvisus* aquaporins except Aqp12 and Bib have an aromatic amino acid (Fig 5). In the second position, Aqp1 and Aqp2 have a histidine, similar to the human aquaporins that only transport water (Fig 5). The human aquaglyceroproteins and the *E*.*coli* Glpf have an alanine or a glycine at this second position (making the pore bigger) [11, 12], whereas the *B*.*improvisus* Glp1 and Glp2 have the slightly larger residue isoleucine (Fig 5). The amino acid at the third position is the least conserved in the constriction site. The pore size is determined by the combined size of all four amino acids at the constriction site and by their structural organization (see the aquaporin 3D models below).

### 3-dimensional structural features of the modeled aquaporins

In order to get 3D structural information of the *B*. *improvisus* aquaporins, *in silico* homology models were built for each of the eight AQP proteins. The water specific human AQP1 (PDB ID 3GD8) and the aquaglyceroporin Glpf from *E*.*coli* (PDB ID 1FX8), were included in the analysis for comparison. The structure models resemble the known common topology for aquaporins with each monomer containing six membrane-spanning helices and two shorter loop-turn-helix segments each containing one of the two NPA motifs. The NPA-loop on the extracellular side partakes in the narrow constriction site together with residues from helices 2 and 5. In the obtained models, the pore radius at the constriction site in each of the AQPs was determined.

**Fig 6:**
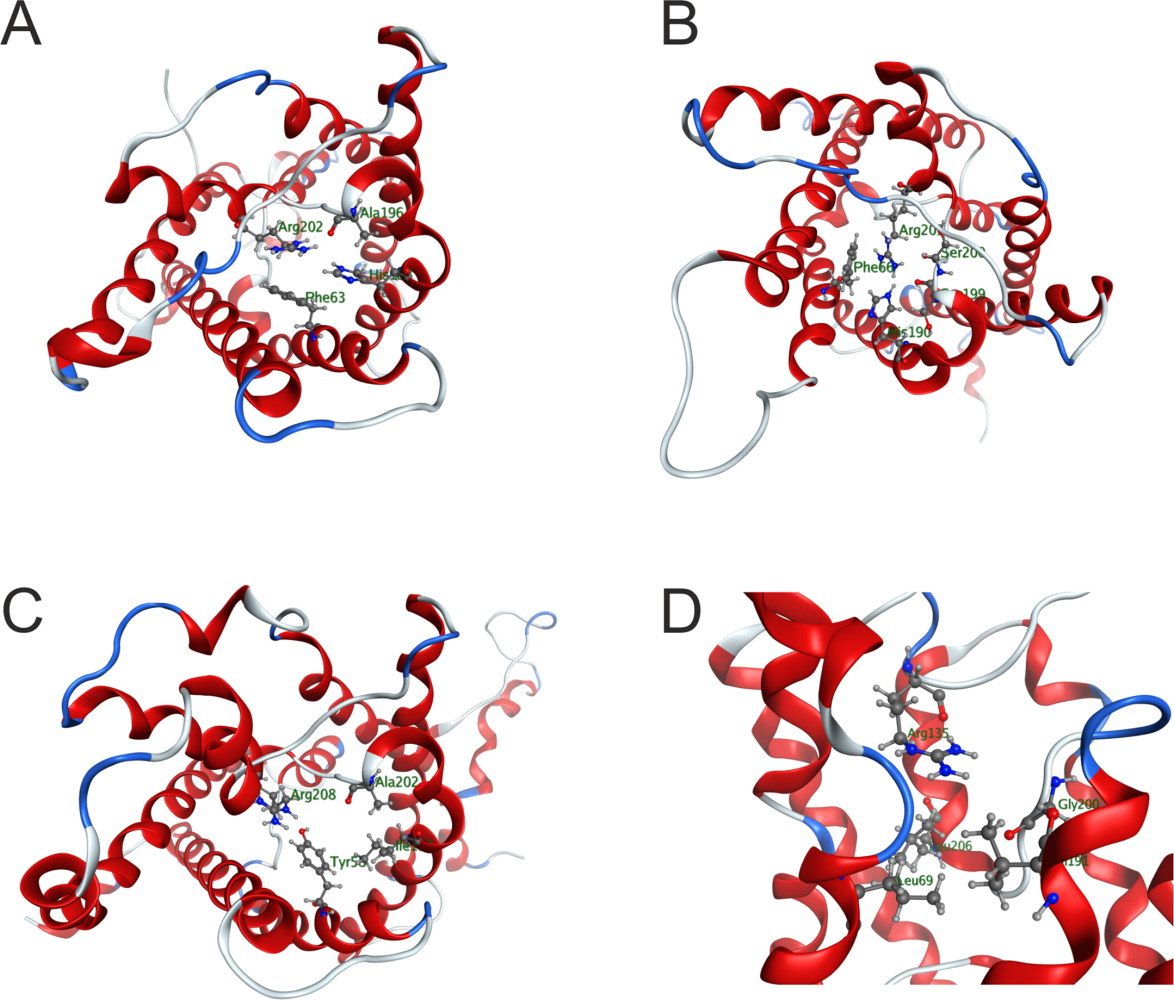
The Ar/R constriction sites in the 3D homology models of the *B*. *improvisus* aquaporins. A-C: The aquaporins are viewed from the extracellular side and the four residues of the constriction site are shown as a ball-and-stick model. A) Aqp1; B) Aqp2; C) Glp2. D: Side view of the Aqp12 constriction site, showing the protrusion of R135 into the pore at the extracellular entrance.

We found that Aqp1 displays a narrow constriction site, caused by hydrogen bonding interactions between His187, Arg202 and the backbone carbonyl of Ala196, as well as π-cation interaction between Arg202 and Phe63 (Fig 6A). The pore radius of the barnacle Aqp1 at the constriction site (~1.6 Å) is roughly similar to that computed for the water transporting human aquaporin AQP1 (~1.8 Å). Aqp2 has a slightly wider pore than Aqp1 at the constriction site (radius ~2.1 Å). The residues being part of the constriction site are highly similar to those in Aqp1, except that the backbone carbonyl in Aqp2 is contributed from a serine (Ser199) instead of alanine (Ala196). In addition, in Aqp2 also the backbone carbonyl Ser200 is present at the constriction site (Fig 6B).

The architecture of the constriction sites of Glp1 and Glp2 differ from Aqp1/Aqp2, in that a tyrosine and an isoleucine partakes together with arginine and alanine, instead of the phenylalanine -histidine pair as indicated for Aqp1/2. For Glp1, a narrow pore is noted (r ~1.6 Å), whereas that for Glp2 is much wider, around 2.7 Å, and in that sense resembles the wider pore diameter of other aquaglyceroporins (Fig 6C). For the *E*. *coli* Glpf a radius of 2.8–3.0 Å was determined. The narrow pore of *B*. *improvisus* Glp1 is the result of π-cation interaction between Arg207 and Tyr58, which pushes the tyrosine towards the center of the constriction site.

*In silico* analyses of the constriction sites in the 3D structure of Aqp12, big brain (Bib) and the big brain-like proteins (BibL1 and BibL2) indicate that these aquaporins might not transport any solutes. In Aqp12, the entrance to the constriction site on the extracellular side is efficiently blocked by an arginine, Arg135 (Fig 6D). However, another possible pore with a pore size of about 2 Å that might facilitate transport was identified on the side of the constriction site. The pore radii at the constriction site of the big brain and the big brain-like proteins were around 3 Å, 1.8 Å and 1.2 Å for Bib, BibL1 and BibL2, respectively. However, in all three of these proteins one or two tyrosine residues placed just below the constriction site protrude into to the pore and may at least partially block transport (S5 Fig).

### Functional analysis of Aqp1, Glp1 and Glp2

**Fig 7:**
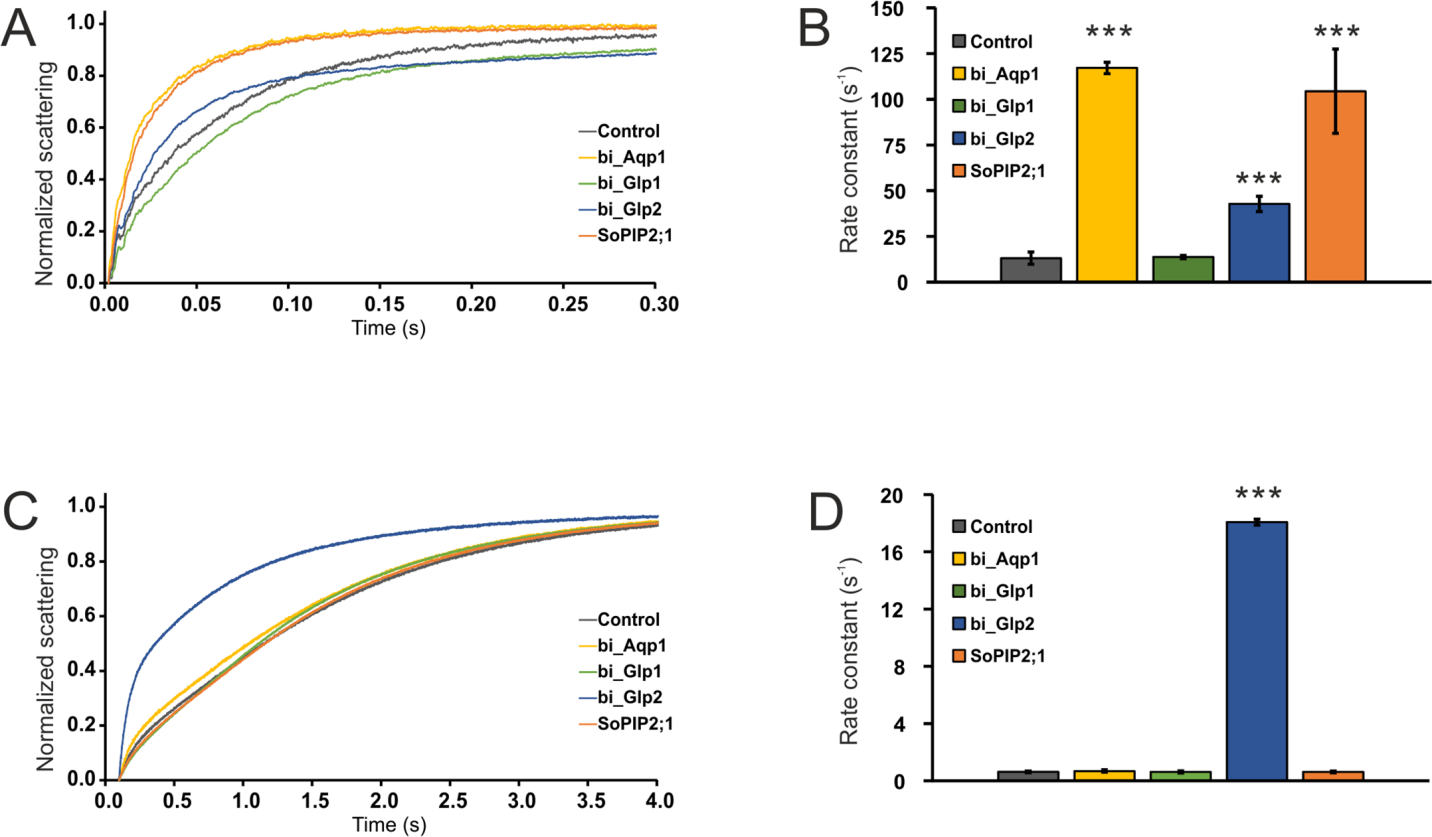
Functional analysis of AQP proteins. The *B*. *improvisus* aquaporins Aqp1, Glp1 and Glp2 were heterologously expressed in the yeast *P*. *pastoris*, purified and reconstituted into liposomes. The spinach aquaporin SoPIP2;1 was included as control. The transport of water and glycerol out of the proteoliposome vesicles were measured as light scattering (indicates liposome swelling/shrinkage) using stopped flow spectroscopy measured at 436 nm. For each experiment, at least three traces were averaged and fitted to either single or double exponential functions using the method of least squares. One typical stopped flow spectroscopy trace is displayed. Reported rate constants are an average of three independent measurements. Error bars show standard deviation.*** = significantly different from control (ANOVA, p<0.001). A-B) water transport. C-D) glycerol transport. The y-axis in B and D shows the rate constant for the curve fitted to the data.

In order to confirm functional classification based on sequence analysis and 3D modeling, his-tagged Aqp1_v1, Glp1 and Glp2 proteins were expressed in the yeast *P*. *pastoris*, purified and reconstituted into liposomes. Water and glycerol transport over the liposome membrane were then measured using a stopped-flow device. As a positive control for water-transport, the well-characterized spinach aquaporin SoPiP2;1 was included [42]. As expected from the phylogenetic analysis and 3D modeling, Aqp1 transported water, with a transport rate about nine times higher compared to the empty liposome control (ANOVA, P<0.001) (Fig 7A and 7B). No glycerol transport was detected for Aqp1 (Fig 7C and 7D). Also, as expected, Glp2 transported both glycerol and water with transports rates of about 29 times and 3 times higher than the control, respectively (ANOVA, P<0.001) (Fig 7A–7D). However, Glp1 did not seem to have any transport at all, neither for glycerol nor for water (Fig 7A–7D). The poor transport of glycerol could be explained from the modeling studies described above, where Glp1 has a narrow pore, which would prevent glycerol transport. However, the lack of water transport by Glp1 could be due to an incorrectly folded protein. Another possible explanation is that Glp1 requires some unknown parameter for its function, such as phosphorylation, ligands, or an alternative pH.

### Differential expression of aquaporins

**Fig 8:**
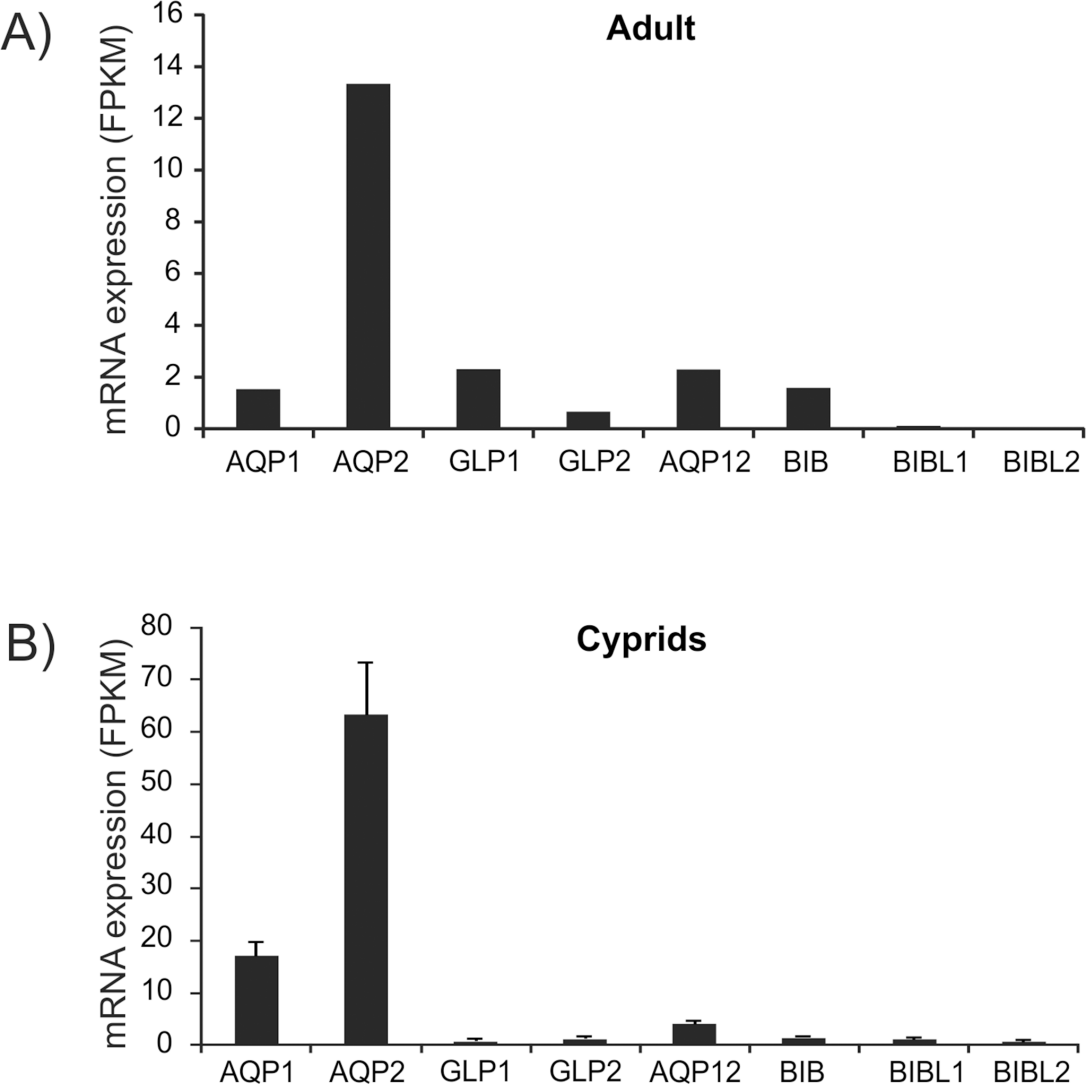
mRNAexpression of aquaporins in *B*. *improvisus*. The mRNA expression of the *B*. *improvisus* aquaporins was determined by RNA-seq in an adult (A) and in cyprid larvae (B) cultivated at seawater salinity (≈ 30 PSU). Total RNA was prepared and sequenced by paired-end Illumina sequencing. Normalized expression levels for the aquaporins were estimated by mapping reads to the aquaporins ORFs using the program RSEM. The expression levels for the different AQP genes are shown as FPKM (Fragments Per Kilobase of transcript per Million mapped reads) for the adult and TMM-normalized FPKM for cyprids. The sample sizes were n = 1 for the adult and n = 4 for the cyprids, with 300 cyprids pooled in four independent replicates. Error bars for the cyprid batches in B shows standard deviation. AQP2 was significantly higher expressed in cyprids compared to all the other aquaporins (ANOVA, P<0.001; see supplementary S1 Table for significant changes for all pair-wise comparisons). Absolute expression values should not be compared between life stages due to lack of normalization between the life stages.

The expression levels of aquaporins in an organism might change through different developmental and ontological stages [22, 43]. In order to analyze the relative expression of the different aquaporins in two life stages of barnacles (cyprid larvae and adults, both from seawater salinity of 30–33 PSU), RNA-seq analyses were performed by mapping reads against the different aquaporin sequences. The *AQP2* mRNA was the most highly expressed aquaporin transcript in both cyprids and adults (Fig 8A and 8B). The expression ratios between aquaporin paralogs within a particular life stage differed between the two life stages, and in particular the expression of the aquaglyceroporins compared to the classical water aquaporins seemed to be somewhat higher in the adult (Fig 8A and 8B). For the BIB and the BIB-like aquaporins, *BIB* seemed to be the dominant form expressed in adults where the expression of *BIBL1* and *BIBL2* was hardly detected, while in the cyprids the expression levels of these aquaporins were roughly similar (Fig 8A and 8B).

**Fig 9:**
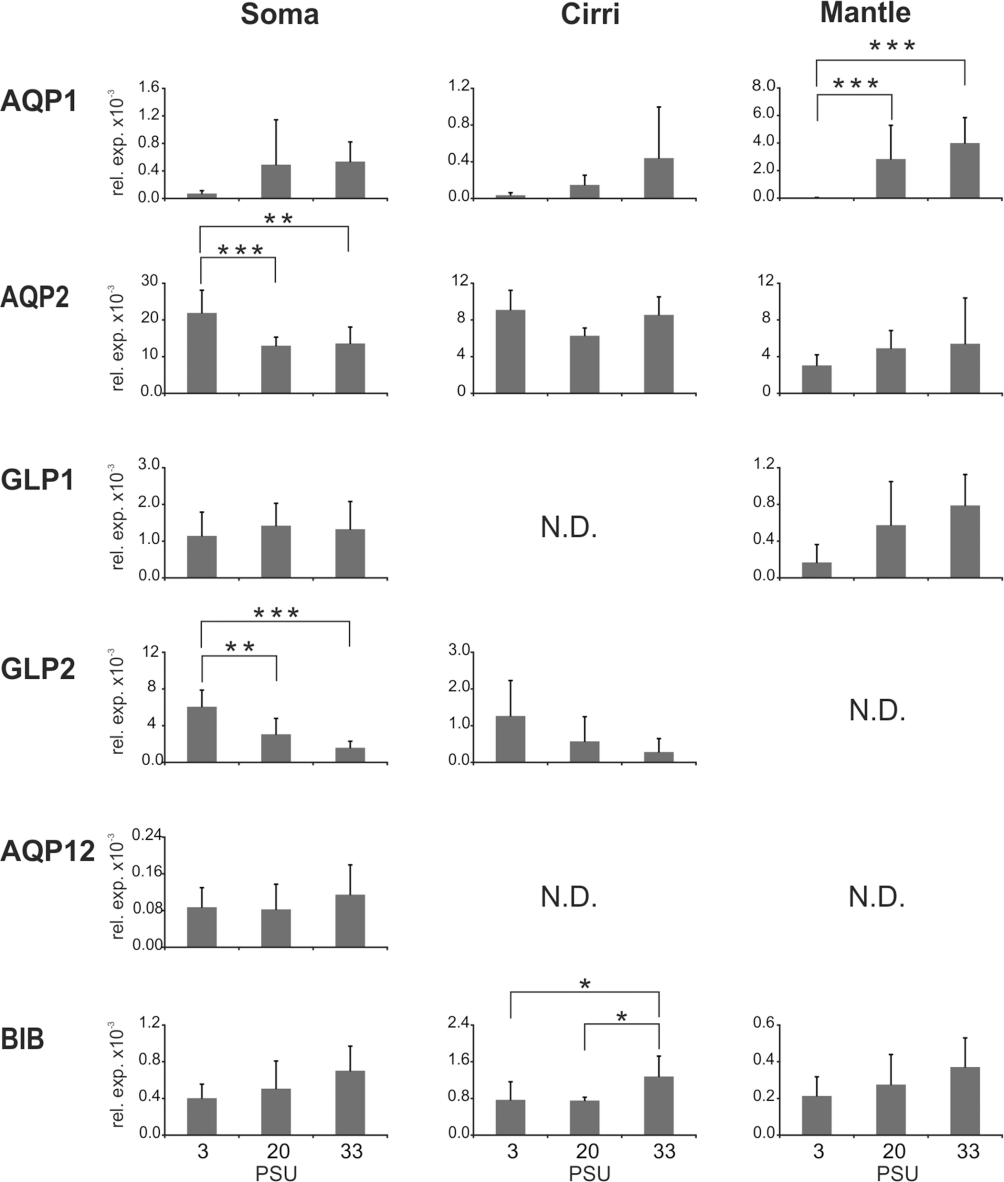
Expression of aquaporins of *B*. *improvisus* in adults during exposure to various salinities. Adult individuals were incubated at three different salinities for 14 days (3, 20 and 33 PSU). For RNA preparation, soma, cirri and mantle of the adults were separated. For each salinity, the tissues (soma, cirri or mantle) from eighteen adults were pooled three-by-three to give six independent samples (n = 6). Quantitative PCR was used to determined aquaporin expression levels relative to actin. In case of cirri at 20 and 33 PSU only 5 independent samples were used in the qPCR due to very low RNA amounts obtained from one of the samples in each case. For some of the samples the expression was below the level of detection (N.D., not detected). Asterisks indicate significant levels (ANOVA): *** p<0.001, ** p<0.01, * p<0.05. Error bars show standard deviation.

To investigate if the *B*. *improvisus* aquaporins might be involved in osmoregulation we examined the mRNA expression changes using qPCR on adult barnacles acclimated for two weeks to three different salinities representing fully marine conditions (33 PSU), brackish water (20 PSU) and extreme low salinity (3 PSU). The genetic variation in *B*. *improvisus* is large (around 3–4%; manuscript in preparation). In order to minimize the effect of PCR-dependent biases resulting from sequence differences between different individuals, we collected and analyzed 18 adults from each salinity treatment and tissues from these individuals were pooled three-by-three for the final analysis (n = 6). Three types of tissues were collected for analysis from each individual; i.e. soma (main body), cirri and mantle. Interestingly, we found that several of the aquaporins displayed changes in expression in relation to the surrounding salinity (Fig 9). In particular, both classical water-transporting aquaporins (*AQP1* and *AQP2*), exhibited significant mRNA expression changes in relation to salinity. *AQP1* showed a drastically decreased expression in the mantle at the low salinity (3 PSU), while the expression of *AQP2* instead was slightly increased in the soma (1.6 fold). However, the magnitude of the expression change was much greater for *AQP1*, with a 121-fold reduction at 3 PSU compared to 33 PSU.

In addition, the aquaglyceroporin *GLP2* responded to changes in salinity with an increased expression of about five-fold in the soma at the lower salinity (3 PSU). *GLP2* expression in the mantle was under the detection level in our assay. *BIB* also displayed a significant change in expression in relation to salinity; its expression was slightly (about 2-fold) reduced in cirri at low salinity. The expression of *BIBL1* and *BIBL2* was below the level of detection in our assay. For the unorthodox *AQP12*, no significant change was seen in the soma and its expression level was under the detection levels in the two other tissues analyzed.

Our data also indicated that some of the AQP genes were differentially expressed in the various tissues (S6 Fig). Interestingly, we found that at seawater salinity (33 PSU), *AQP1* was significantly higher expressed in the mantle than in the other two tissues (more than 7-fold higher). In contrast, the expression of the paralog *AQP2* was high in both soma and cirri, and relatively low in the mantle. Also the aquaglyceroporins *GLP1* and *GLP2* displayed higher expression in soma compared to the other tissues. *BIB* was the only aquaporin that had a significantly higher expression in cirri compared to the other two analyzed tissues.

## Discussion

### Evolution of aquaporins in crustaceans

We here identify, for the first time, the whole repertoire of aquaporins in a barnacle, the euryhaline species *B*. *improvisus*. The knowledge of aquaporins in crustaceans is limited and the complete aquaporin repertoire is currently only available for the salmon louse *L*. *salmonis* [22] and the water flea *D*. *pulex* (sequences in the NCBI NR database). Barnacles belong to the crustacean subclass Thecostraca (infraclass Cirripedia), and are evolutionary quite far from both the copepod *L*. *salmonis* and the branchiopod *D*. *pulex* with the last common ancestors estimated to about 400 and 500 million years ago, respectively [44]. Thus, our study of the aquaporin repertoire of *B*.*improvisus* provides valuable information about the representation and function of aquaporins in Thecostraca, and deepens our understanding of the evolution of aquaporins in Crustacea.

The subfamilies of aquaporins in *B*. *improvisus* are the same as those found in salmon louse and *Daphnia*, with the exception that *B*. *improvisus* has the here identified big brain-like group (BibL1 and BibL2) not present in the other two species. The number of members in each aquaporin subfamily also differs. While both *Daphnia* and salmon louse have only one water-specific classical aquaporin, *B*. *improvisus* has two (Aqp1 and Aqp2). In the case of aquaglyceroporins, *B*. *improvisus* has two (Glp1 and Glp2), while salmon louse has three and *Daphnia* has six. Unorthodox Aqp12 is found in one copy in both *B*. *improvisus* and *Daphnia* as in most other arthropods, while salmon louse is unusual in having two paralogs [22]. We found that *B*. *improvisus* has one BIB and two BIB-like proteins, while salmon louse, *Daphnia*, *Drosophila* and most other sequenced arthropods have only BIB. Among insects, only the mosquito *Anopheles gambiae* seems to have two big brain paralogs [4], however, both of these belong to the BIB clade (Fig 1, S1A and S1B Fig). Lastly, phylogenetic analysis of the *B*. *improvisus* aquaporins shows that the gene-pairs *AQP1*/*AQP2*, *GLP1*/*GLP2* and *BIBL1*/*BIBL2* are each found on a separate evolutionary branch (Fig 1, S1A and S1B Fig) indicating quite recent duplications. The exon/intron structure also supports this; each pair share the same exon/intron borders and phasing.

### *B*. *improvisus* has a big brain-like group of aquaporins

We found that in addition to Bib, *B*. *improvisus* has two BIB-like aquaporins constituting a subfamily of proteins not found in other sequenced crustacean species. However, phylogenetic analyses show that some chelicerate species also have aquaporins of both the big brain and big brain-like groups (Fig 1 and S1 Fig). Interestingly, the horseshoe crab *Limulus polyphemus* potentially has as many as eight BIB homologs, six of which belong to the BIB clade and two to the BIB-like clade (S1 Fig). A number of recent investigations have provided evidence for one or more whole-genome duplication events in some species of Chelicerata, e.g. in *L*. *polyphemus* [45, 46], that could partly explain its high number of BIB paralogs. Besides *L*. *polyphemus*, other chelicerate species such as the spiders *Parasteatoda tepidariorum* and *Stegodyphus mimosarum*, have proteins belonging to both the BIB and BIB-like clades. Taken together this indicates that the formation of the BIB-like proteins is a rather ancient event with a loss of the BIB-like proteins during the evolution of the crustaceans *Daphnia* and salmon louse, while still being present in *B*. *improvisus* and some chelicerate species. Other crustaceans than *B*. *improvisus* may also have BIB-like proteins, but they could be lowly expressed as in *B*. *improvisus* and therefore undetectable in available RNA data sets; information on the complete aquaporin repertoire in crustaceans will have to await more genome sequences.

Predicting the function for the BIB-like proteins is difficult, since even for BIB the function is largely unknown. A mutation in the big brain gene in *Drosophila* has shown that it is important for neural development in the embryonic brain [47], and potentially the BIB-like proteins are also involved in the development/functioning of the nervous system. The *Drosophila* big brain aquaporin has been reported not to transport water, which is in line with our *in silico* 3D homology model of the *B*. *improvisus* Bib (S5A Fig) and with a homology model of the big brain protein from the yellow fever mosquito *Aedes aegypti* [48]; in both models tyrosines partially block the transporting pore. Tyrosines appear to partially block the transporting pore also in our models of BibL1 and BibL2, (S5B and S5C Fig), suggesting that the BIB-like proteins also do not transport water. The *Drosophila* Bib is reported to have ion-conductance and cell-adhesion properties [6, 7], which could be the case also for the BIB-like proteins. These properties could indicate a possible role in osmoregulation, even if no water transport occurs via these proteins. In addition, BibL1 and BibL2 might be involved in biological processes that are more important in barnacles than in *Daphnia* and salmon louse, which lack BIB-like proteins. Future experimental studies will have to be conducted in order unravel the true function of BibL1 and BibL2.

### *AQP1* and *AQP2* are expressed as splice variants

N-terminal splice variants were reported for the salmon louse GLPs [22]. In *B*. *improvisus*, however, we could not find any splice variants of GLPs, but instead found evidence for splicing in the C-terminal region of the water aquaporins Aqp1 and Aqp2. Transcript sequences for a water-specific classical aquaporin from the crustacean *Gammarus chevreuxi* (NCBI TSA database; HADC01025558.1, HADC01025557.1) also indicate the existence of two C-terminal splice variants, most likely created by a similar splicing mechanism as occurs for the *B*. *improvisus AQP1* and *AQP2* transcripts (exclusion of exon 5). A conserved splicing mechanism in two evolutionary divergent species might indicate that the splice variants have some important function. C-terminal splice variants of an Aqp1-like protein has also been found in the insect *A*. *gambiae*, where one of the splice variants is specifically expressed in the adult female ovary [49]. The cytosolic C-termini of aquaporins have been shown to be involved in intracellular trafficking of the aquaporins and also to interact with different proteins [50, 51]. In our 3D-homology models, the overall structures of the longer splice variants for *AQP1* and *AQP2* (Aqp1_v2 and Aqp2_v2) are quite similar to their shorter counterparts (Aqp1_v1 and Aqp2_v1), but the positions in space of their longer C-termini deviate from the C-termini of the shorter variants (S7 Fig). The different splice variants of *AQP1* and *AQP2* might be expressed in different tissues and/or have different functions, potentially resulting from interaction with different proteins.

### Aquaporins and their potential involvement in osmoregulation

The role of AQPs in salinity acclimation has been most well studied in fish, where it has been shown that expression of aquaporins in osmoregulatory organs are responsive to changes in salinity [52]. For example, the expression levels of many fish AQP paralogs are up-regulated in the intestine of fish acclimated to seawater [52]. Fish in seawater drink large amounts of water to achieve an active fluid absorption through ion-coupled water uptake. As the paracellular permeability of the intestinal epithelium has been shown to decrease after seawater acclimation, the increased intestinal fluid absorption occurring under these conditions is suggested to mainly use a transcellular route through AQPs [53].

Few earlier studies have investigated the osmoregulatory role of AQPs in crustaceans. An Aqp1-like aquaporin in the blue crab *C*. *sapidus* was shown to be transcriptionally up-regulated during exposure to low salinity [20]. In the shrimp *Litopenaeus vannamei*, expression of an aquaporin was also up-regulated at low salinity conditions [21]. In contrast, down-regulation of aquaporins was shown in the swimming crab *P*. *trituberculatus*, where the mRNA expression levels of AQPs decreased under both hypo-osmotic and hyper-osmotic stress conditions [19]. We found that in *B*. *improvisus*, the relative abundance of aquaglyceroporins compared to water-specific aquaporins was higher in the adult compared to cyprids, perhaps indicating that glycerol transport is of greater importance for adults. Aquaglyceroporins could be functional in anti-freeze mechanisms for the sessile adult stage that probably needs to increase and control glycerol content in various tissues during winter to avoid freezing [54, 55].

Most importantly, we report that when adult animals of *B*. *improvisus* were subjected to low salinity, *AQP1* expression was substantially down-regulated in the mantle. This might be a mechanism to reduce the osmotically driven transcellular uptake of water in low-salinity environments. The mantle surrounds the main body (soma) and is thus the tissue in direct contact with the surrounding water. The mantle also provides a large surface area making this tissue a plausible site for osmoregulation in barnacles. It was shown recently that adults of *Balanus amphitrite* has a mantle epithelium that is rich in cells that produce an electrolyte-rich secretion and display indications of chloride transport [56]. Similarly, also epithelia of nauplii and cyprids were suggested to transport chloride and to contain cells rich in mitochondria. It was proposed that these cells might be involved in osmoregulation at low salinities [56]. It could be speculated that barnacles in all life stages distribute osmoregulation over the whole body so that most/all epithelial cells in contact with the external salinity take part. In the future, it would be valuable to perform immunohistochemistry or *in situ* hybridization to investigate the spatial expression of the *B*. *improvisus* aquaporin paralogs in both larvae and adults exposed to varying salinity, in order to better understand their specific biological functions and to disclose osmoregulatory tissues.

## Concluding remark

Our analyses reveal that *B*. *improvisus* contains eight genes for aquaporins, where two of them, *BIBL1* and *BIBL2*, encode proteins that constitute a new group of aquaporins not yet described in arthropods. In addition, we found that the expression of some of the aquaporins are regulated in response to salinity changes. In particular, the two paralogs of classical water transporting aquaporins display very different responses with *AQP1* expression being almost fully repressed at low-salinity conditions. The *AQP1*-regulation is mainly apparent in the mantle, which is interesting given that the mantle earlier has been proposed as a site for osmoregulation in barnacles. *B*. *improvisus* is a truly brackish species that is well adapted to establish itself in low-salinity environments, enabling invasion of brackish waters like the Baltic sea where it was quite recently introduced (<200 years ago) [28]. Scenarios of global climate changes predict future enhanced rainfall leading to freshening of salty environments; the Baltic Sea is predicted an up to 50% salinity decrease by 2100; [57]. This will influence the success of *B*. *improvisus*, potentially leading to competitive exclusion of native species [58, 59]. To what extent the aquaporin repertoire in *B*. *improvisus* is unique and how it possibly plays a role in the extreme salinity tolerance of this species and in its preference for brackish environments is unclear. A better understanding of this has to await more mechanistic experimental data as well as comparative analysis of other barnacle species with varying salinity tolerance.

## Material and methods

### Genome and transcriptome sequencing

Maintenance of laboratory cultures of adults of *B*. *improvisus* and the rearing of cyprids were as earlier described, but no antibiotics were used [60, 61]. The barnacle cultures were used for obtaining DNA and RNA for sequencing. High quality genomic DNA was prepared from one adult individual of *B*. *improvisus* (using the same methodology as previously used for cyprids [31]) and sent to the national sequencing facility SciLife Lab (Stockholm) for paired-end Illumina sequencing (2x101 bp read length) of both 150 bp and 300 bp fragment libraries. The reads were filtered and trimmed using the FASTX-Toolkit [62]. Assembly of the sequence reads into contigs was made using the CLC *de novo* assembler v4.06beta.67189 (CLCbio). The sequencing of genomic DNA was part of an ongoing genome project for *B*. *improvisus* [63].

RNA sequences were obtained from two different adults and a batch of hundreds of cyprid larvae. One adult was used for short-read Illumina sequencing and one was used for long-read single molecule PacBio sequencing. Illumina sequencing was also used for the cyprid larvae. High-quality total RNA was prepared from one adult as previously described [31] and sent to SciLifeLab for paired-end Illumina sequencing (2x101 bp read length). For PacBio RNA sequencing the sample was depleted of ribosomal RNA using the ribo-zero Magnetic Gold Kit (Epicentre). Double stranded cDNA was then prepared using the SMARTer PCR cDNA synthesis kit (Clontech). RNA from cyprids was prepared from a batch of roughly 500 cyprids for each of 16 samples [31] and sent for paired-end Illumina sequencing (2x101 bp read length). Assembly of digitally normalized trimmed Illumina reads was done using trinityrnaseq_r2013_08_14 with default parameters. The Pacbio RNA reads were sequenced and assembled in three fractions: 1–2 kb (8 cells), 2–3 kb (24 cells) and 3–6 kb (24 cells). Consensus reads were constructed using the Pacbio IsoSeq pipeline v2.3.0 using a quality value cutoff of 99 for the shorter 1–2 kb and 2–3 kb fractions and 97 in the case of the longest 3–6 kb fraction.

### AQP expression in an adult and cyprids at 33 PSU

Expected read counts for an adult and cyprids cultured at 33 PSU were estimated using RSEM (v. 1.2.7) that leveraged bowtie (v. 0.12.7) against a Trinity (v. trinityrnaseq_r2013_08_14) de novo assembly [64] from adult data where aquaporin contigs were replaced by corresponding, full-length template sequences from other assemblies or PCR clones. FPKM normalized adult expression values were taken directly from RSEM [65] output. The normalized cyprid expression values were obtained by taking the cyprid expected counts from RSEM, subjecting them to independent filtering (>0.1 cpm in at least 8 samples), TMM normalization and RPKM normalization using edgeR (v. 3.4.2) [66, 67]. The weak filtering parameters (although generally not optimal) were required to get any data for the lowest expression aquaporins. Adult and cyprid expression values cannot be directly compared due to e. g. batch effect between life-stages that cannot be estimated due to lack of adult replication and a lack of TMM normalization between the life stages. Batch adjustment for the cyprid dataset using the limma (v. 3.18.13) function removeBatchEffect [68] had negligible effect.

ANOVA analysis of aquaporin expression in cyprids (Fig 8B) was performed on log-transformed FPKM values (raw data were non-normal). Variances between aquaporins were found not to be heterogeneous (by Levene’s test, p>0.05). There was a highly statistically significant effect of gene (different genes had different expression levels, ANOVA, p<0.0005) and individual differences between genes were tested by Tukey’s HSD *post-hoc* test (S1 Table).

### Cloning and sequence verification of the *B*. *improvisus* aquaporins

In order to clone and verify the various AQP sequences obtained from genome and/or transcriptome sequences, total RNA was prepared from a batch of about 500 cyprids and used to prepare cDNA as previously described [31]. The obtained cDNA was used as template for PCR amplification. PCR primers for cloning of the open reading frames of the *B*. *improvisus* aquaporins were designed using sequences from RNA and DNA contigs (S2 Table). In the case of AQP2 and BIBL1, the full-length ORF was not found in the contigs available at the time of cloning, so 5´and 3´ RACE was performed using the generacer kit (Invitrogen). For RACE, a touchdown PCR was first performed, which was followed by a nested PCR using 1 μL of the touch-down PCR product. In all PCRs there is an initial denaturation step for 2 min at either 98°C (when using the polymerase pfuUltra; Stratagene) or 95°C (when using the polymerase Expand high fidelity; Roche) and a final elongation step of 7–10 min at 72°C. During cycling, denaturation was performed at either 98°C (using pfuUltra) or 95°C (using Expand high fidelity) for 30 s and the elongation time was 2–3 min (1 min/1000 bp amplified). For all reactions 35 cycles were run. Annealing was performed for 30 s at the temperature indicated in S2 Table. The touchdown program for RACE contains 5 cycles with annealing at 72°C, 5 cycles with annealing at 70°C and finally 25 cycles with annealing at 65°C. The sequences of all cloned AQPs were determined by Sanger sequencing (Eurofins genomics). The AQP1_v2 and AQP2_v2 C-terminal splice variants of the water aquaporins AQP1 and AQP2 respectively, were not cloned by PCR. For AQP1_v2 the complete open reading frame was obtained by combining an Illumina corrected Pacbio read, spanning from amino acid 41 to the last amino acid (aa 299) of AQP1_v2, with amino acid 1 to 40 of the PCR clone containing the whole open reading frame of AQP1_v1. The sequence for AQP2_v2 was obtained from an assembled Trinity contig, but was also found in the Pacbio sequence analysis. In case of BIBL1, the 5´ RACE failed to give the coding sequence for the first 146 amino acids of the protein. The complete open reading frame for BIBL1 was therefore obtained by combining amino acids 1–146 of a DNA contig covering amino acid 1–310, with a PCR cDNA clone containing the rest of the open reading frame (amino acid 147 to 389). Amino acids 1–146 encoded by the DNA contig were covered by two partly overlapping RNA contigs, showing the lack of intron sequence in this part. Complete open reading frames for all aquaporins are shown in supplementary file S1 AQP sequences.

### Accession numbers for the *B*.*improvisus* aquaporins

The *B*. *improvisus* aquaporin sequences have been deposited to GenBank and have the accessions number KY508284-KY508298.

### Phylogenetic analysis of the *B*. *improvisus* aquaporins

Phylogenetic analysis of aquaporin sequences from insects, crustaceans, humans and chelicerates was performed using maximum likelihood applying PhyML v3.0 [69] or RaxML v8.0.19 [70], and by using Bayesian inference applying MrBayes v3.2 [71]. Sequences were obtained from the NCBI Nr or TSA databases. Aquaporin sequences for *Carcinoscorpius rotundicauda* were downloaded from the database referred to in Kenny et al [46]. A few sequences containing only partial open reading were frames included, however with the criteria that both NPA motifs should be covered. Two partial sequences for the Limulus big brain proteins were however included in which only one NPA motif was present. Accession numbers for all sequences used can be found in S3 Table. Protein sequences were aligned using Muscle v3.8.31 [72] or Muscle 3.7 (for PhyML 3.0). For Fig 1, PhyML analyses were performed at the Phylogeny.fr website, using the WAG substitution matrix and aLRT non-parametric branch support based on a Shimodaira-Hasegawa-like procedure, but without Gblocks. For S1 Fig, MrBayes analyses were done using the mixed amino-acid model and iterated for 25 million generations with 7 heated and 1 cold chain. Probability distributions were analyzed using Tracer v1.6.1. RaxML analysis was performed on the same data used in S1 Fig with PROTGAMMAAUTO for protein model estimation and support values calculated using rapid bootstrap mode for 500 trees (data not shown).

### Heterologous membrane protein overproduction and membrane preparation

The cDNAs for the AQPs to be functionally tested (AQP1_v1, GLP1, GLP2) were fused to a C-terminal 8x histidine tag and cloned into the Sfu-XbaI site of the plasmid pPICZB (Invitrogen). The pPICZB-AQP plasmids were then linearized using PmeI and transformed into the yeast *Pichia pastoris* according to the protocol for the EasySelect Pichia Expression System (Invitrogen). In order to screen for *P*. *pastoris* colonies containing multiple copies of integrated plasmid, the colonies were resuspended in YPD and diluted to an OD of 0.02. 2 μL were then pipetted on plates containing 100 μg/mL, 500 μg/ml (omitted for AQP1), 1,000 μg/mL or 2,000 μg/mL Zeocin respectively. Colonies that managed to grow on 1000 μg/mL Zeocin after 3 days were re-streaked and inoculated into 5 ml Buffered Glycerol Complex Medium (BMGY) and cultured overnight. The yeast cell suspension was centrifuged and the resulting pellet resuspended in Buffered Methanol-Complex Medium (BMMY) to an OD 0.5–0.7 and grown for 6 h. A western blot using anti-His-antibodies (GenScript,THE^TM^ His Tag Antibody, Cat No A00186-100 or Clontech 6xHis Mono Ab, Cat No 631212) was then performed on a membrane preparation from the cultured cells to confirm expression of the aquaporins.

The AQP overproducing strains were grown in a 3 L fermentor following the general guidelines from Invitrogen for protein production in *P*. *pastoris* (Pichia Fermentation Process Guidelines), typically resulting in 200–400 g cells (wet weight)/L culture after 24–48 h of methanol induction. For membrane preparations, yeast cells were resuspended in buffer A (50 mM Tris-HCL, 150 mM NaCl, 0.5 mM PMSF and 1 mM β-mercaptoethanol), homogenized and lysed by passing the yeast slurry either twice through a French press or by using a Beadbeater. Thereafter debris and unbroken cells were removed by centrifugation at 5,000 x g, 4°C, for 45 min and the membranes were collected by ultracentrifugation (158,000 x g, 90 min, 4°C). Glp1 and Glp2 membranes were dissolved in buffer A and stored at -80°C until further use, whereas the membranes containing Aqp1 were washed two times, first in Urea buffer (4M Urea, 50 mM Tris-Hcl, pH 9.5, 2 mM EDTA, 2 mM EGTA) and then in 20 mM NaOH. Finally, Aqp1 membranes were resuspended in resuspension buffer (25 mM Tris/HCl, pH 7.5, 250 mM NaCl, 10% Glycerol, 1mM β-MeOH) and stored at -80°C until further use. The final concentration of membrane proteins was 10–30 mg/ml buffer. A western blot of Aqp protein containing extracts of the different purification steps are shown in S8 Fig.

### Solubilization and purification of membrane proteins (Aqp1_v1, Glp1 and Glp2)

Membranes containing Aqp1_v1 were solubilized in resuspension buffer over-night at 10°C by drop-wise addition of LDAO to a final concentration of 0.5%. Unsolubilized material was spun down at 180,000 x g for 30 min at 4°C and the supernatant filtered through 0.45 μm. The crude protein mixture was looped over a HisTrap HP (GE Healthcare) column Ni-NTA affinity column, pre-equilibrated with resuspension buffer + 0.1% LDAO for 2 h. Unbound material was washed away with 40 mM imidazole, and Aqp1_v1 was eluted with 400 mM imidazole. Protein fractions were pooled and concentrated using a Vivaspin 10K MWCO (Sartorius Stedim Biotech GmbH). The concentrated protein solution was filtered through a 0.2 μm filter and injected onto a Superdex 200 Increase gel filtration column (GE Healthcare) equilibrated with 20 mM Tris/HCl pH 7.5, 100 mM NaCl, 0.1% LDAO. Pure Aqp1_v1 was pooled and concentrated as described above. Membranes containing Glp1 or Glp2 were solubilized with 1.5% DM in a buffer containing 300 mM NaCl (Glp1) or 80 mM NaCl (Glp2) and 50 mM TrisHCl pH 7.4 for 3 h at RT on a rolling table. Non-solubilized material was removed by ultracentrifugation at 138,000 x g for 30 min at 4°C. To purify the protein, the supernatant containing the solubilized protein was incubated with 4.5 ml nickel gel slurry (Qiagen) over night at 4°C. The unbound protein was removed by centrifugation at 3,500 g for 4 min. The resulting gel-slurry containing the bound protein was washed with elution buffer (40 mM tris pH 7.4, 300mM NaCl, 0.3% DM, 10% glycerol) containing 40 mM imidazole and then transferred to a gravity flow column. Elution was performed in 1 ml fractions using elution buffer containing step-wise increasing amounts of imidazole (2x40 mM, 4x80 mM, 5x250 mM and 5x400 mM).

The fractions containing substantial amounts of pure AQPs as shown by western blot and Coomassie staining (second fraction of 250 mM up to last fraction of 400 mM imidazole) were pooled (S9 Fig), desalted using a desalting column and concentrated using a Vivaspin 10K MWCO column. The resulting protein concentrations (typically 15 mg/mL) were calculated using the theoretical extinction coefficient for the different aquaporin proteins and the A260 value obtained from NanoDrop™ measurements.

### Preparation of proteoliposomes

Liposomes were created from *E*. *coli* polar lipid extract (Avanti Polar Lipids) by slow detergent removal using BioBeads SM-2 (BioRad). A 25 mg/mL stock solution of *E*. *coli* polar lipids was created by suspending the lipids in reconstitution buffer (50 mM NaCl, 50 mM Tris-HCl pH 8.0). Lipids were diluted to 4 mg/mL in reconstitution buffer and β-OG was added to a final concentration of 2% (w/v). Protein was added to a theoretical lipid-to-protein ratio (LPR) of 50 and the solutions were briefly incubated on ice. Biobeads were then added to a bead-to-detergent weight ratio of 30:1 and the pre-liposome mixtures were incubated at 10°C with slight agitation overnight. The mixtures were centrifuged for 30 minutes at 138,000 x g. The supernatant was discarded and the pellet slowly resuspended to 2 mg lipid/mL using either reconstitution buffer or reconstitution buffer supplemented with glycerol (ca 580 mOsm, for the analysis of glycerol transport), and then filtered through a 0.2 μm filter. The liposome samples were allowed to rest for at least an hour at room temperature before measurements. A western blot was performed to ensure incorporation of the aquaporins into the liposomes (S10 Fig).

### Water and glycerol activity assays of AQPs

The osmotic water and glycerol permeability of the vesicles were measured using stopped flow spectroscopy (μSFM-20, BioLogic Science Instruments). 74 μL liposome solution and 74 μL sucrose solution were mixed and the light scattering at 90° was measured at 436 nm for 4 s (water transport) or 20 s (glycerol transport). For water transport assays the sucrose solution was 280 mM while for the glycerol assays the sucrose solution was adjusted to have the same osmolality as the glycerol-liposome solution, ca 580 mOsm. For each experiment at least three traces were averaged and fitted to either single (control liposomes or proteoliposomes with low- or non-transporting protein) or double (proteoliposomes) exponential functions using least squares and the rate constant were extracted. In the case of double exponential functions being fitted, the slower of the two rate constants is assumed to represent the passive diffusion through the liposomal membrane, while the significantly larger rate constant represents the diffusion through aquaporin channels. The single and double exponential functions are "A1 * e^(-k1*t) + A2" and "B1 * e^(-k1*t) + B2 * e^(-k2*t) + B3", respectively, where k1 and k2 are the rate constants. Note that A1, B1 and B2 in our case have negative values. Reported rate constants are means (±SD) of at least three independent measurements.

Statistical analyses of the transport data were performed separately for water and glycerol transport (Fig 7B and 7D). ANOVA analysis was performed on log-transformed rate values (raw data were non-normal). Variances between aquaporins were found not to be heterogeneous for glycerol transport while being heterogenous for water transport data (by Levene’s test, p>0.05). This means that the statistical analysis on water transport should be viewed with somewhat more caution. There was a highly statistically significant effect of aquaporin (different aquaporins had different transport rates, ANOVA, p<0.0005) and individual differences between aquaporins were tested by Tukey’s HSD *post-hoc* test.

### *In silico* homology modeling of AQP 3D structures

To obtain the 3D structures of all barnacle aquaporins, the respective amino acid sequences (Fig 4) were employed for *in silico* homology modeling using the YASARA software [73, 74]. Homology modeling relies on the fact that the tertiary structure and folding of two proteins are similar if the sequences are related [75, 76]. By identification of one or more known protein structures which amino acid sequences resemble those of the barnacle AQP amino acid sequence, 3D models of the latter can be constructed *in silico*.

Considering the fact that the higher the percentage of identical residues the better is the 3D model, a BLAST search [77] was first performed of the protein databank (PDB) [78] to identify suitable templates with well-established experimental structures. Alternative alignments were created for each template using a stochastic approach [79] and the models built from these. Missing loops and side-chains were modeled by optimizing against a large number of conformations, and subjected to combined modeling utilizing steepest descent and simulated annealing minimization with backbone atoms kept fixed. Finally, a fully unrestrained simulated annealing minimization was run for the entire model. Details regarding the settings used in YASARA are included in S4 Table.

A number of possible templates were identified that were used to extract a position-specific scoring matrix (PSSM) where after the PDB was searched for a match (*i*.*e*. hits with an E-value below the homology modeling cutoff defined; see S4 Table for details). Out of the many identified possible templates for each aquaporin, YASARA selected the five best hits for each, based on BLAST E-value and alignment score, which were used as templates to build fifteen 3D models. Aiming to improve the accuracy of the final AQP model, further refinement was achieved combining the best parts of different models, *i*.*e*. lower quality regions of the top scoring model or missing parts at C- and N-termini were iteratively replaced with corresponding high-quality fragments from other models. Once the homology modeling was finished, the program assessed the quality of the derived AQP models through the estimation of weighted averages of the individual Z-scores, based on a combination of dihedral angles, 1D packing and 3D packing. This procedure captures both correctness of the backbone and side chain dihedrals, and packing interactions. The PDB entries of the main template and fragments added for improved final models are listed in S5 Table. The final 3D model of each AQP (for the complete models see supplementary files S1–S10 Models) was then used in analyses of pore structures and various other properties, as outlined below.

As seen in S5 Table, different templates were employed in the homology modeling, and the selection of aquaporin templates clearly groups Aqp1, Aqp2, Bib, BibL1 and BibL2 into one set, Glp1 and Glp2 into a second set, and Aqp12 as a separate entity altogether. Human aquaporins 2, 4 and 5, the spinach aquaporin SoPIP2;1 and the eye lens aquaporin AQP0 are the most common templates for the first set, with human aquaporin 4 (PDB ID 3GD8) being the main template in the majority of cases. For the second set, the templates are instead largely found among aquaglyceroporins, with the main template being Glpf either with (Glp1) or without (Glp2) the glycerol substrate present in the template. For Aqp12, the templates are primarily different mutants of aquaporin Aqpz without or with mercury.

### *In silico* estimation of molecular surfaces and pore radii of AQP

The software Molecular Operating Environment (MOE) [80] was used to compute molecular surfaces of the aquaporins, onto which electrostatic properties and hydrophobicity/hydrophilicity were mapped. MOE was also utilized to depict residues of the constriction sites and NPA loops.

Measurement of pore diameters was performed using the ChExVis software [81], by uploading the generated models to the ChExVis server [82]. ChExVis uses a combination of Voronoi diagrams and Delauney triangulation between atoms based on their van der Waals radii to identify pores and tunnels in proteins. Besides the radius along the progression of the pore, various properties averaged along the channel diameter can also be computed and displayed as function of distance. Determination of pores and their radii is not entirely straightforward, and in some instances additional paths were detected by the ChExVis software, that display a slightly different amino acid lining at possible ‘branching points’. The performance of different software for pore determination has previously been reported [81, 83], showing the complexity of pore determination.

Besides the homology models derived from the barnacle AQPs identified in the current work, the same analysis was also performed on the human aquaporin AQP1 [11] and aquaglyceroporin Glpf [12]. The aquaporins form tetrameric structures, and all measurements on pore diameters were throughout made on monomer unit A.

### Exposure of *B*. *improvisus* adults to different salinities

To study the mRNA expression changes for AQPs in relation to salinity, adult barnacles (*B*. *improvisus*) were collected on stones from the shallow parts of the Hvaler archipelago, Norway (59°9’9”N, 11°11’15”E) on November 5, 2012. The stones with adult barnacles were directly transported in seawater from the collection site (17 PSU) to Sven Lovén Centre for Marine Sciences—Tjärnö (Strömstad, Sweden) where they were placed in separate aquaria (one stone with multiple barnacles in each aquarium, 18 aquaria in total) within a large re-circulating water system (approx. 200 L) set at a salinity of 20 PSU and left to acclimatize for two weeks (see further description of experimental system below). The barnacles were fed with newly hatched *Artemia* approximately every third day.

For the experiment, three large independent recirculating systems (170 L each) were used, each including 6 aquaria (6 L each). The flow through each aquarium was 20-25L/h. Each re-circulating system was set to three different salinities (3 PSU, 20 PSU and 33 PSU), which was obtained by mixing filtered (0.2 μm) deep seawater from the Kosterfjord (30–34 PSU) with filtered tap water. The salinity treatments were selected based on the natural range at which *B*. *improvisus* is found—a species known for broad tolerance to different salinities [23–25, 28]. Water quality variables including temperature, pH and salinity were monitored routinely during the experiment. The experiment was maintained at 19°C and a light regime of 14:10 h (L:D).

After 2 weeks acclimation, six stones with multiple barnacles (ca 50–500 individuals per stone) were placed in individual aquaria within each of the three re-circulating systems with different salinities. Observations made directly after transfer to altered salinities showed that most barnacles re-gained cirral activity within few minutes, even in the high and low salinity treatments (the delay was slightly longer in the 3 PSU treatment).

After two weeks (day 14), the stones with barnacles, which had been exposed to different salinities, were placed in cooling boxes with treatment water, and transported to Gothenburg for further sampling of RNA.

### RNA preparation for qPCR-based quantification

After exposure for 14 days at 3, 20 or 33 PSU, 18 adults for each salinity were removed from the stones using a scalpel, put into RNA later^TM^ (Qiagen) and stored at -80°C. For RNA preparation, the individual barnacles were thawed and the soma, cirri and mantle were dissected out using a scalpel. For each salinity, the specific tissues from three individuals were pooled leading to six sample replicates for each tissue-type and salinity. Tissues from individuals were pooled in order to get enough material and to reduce the effects of sequence variation among individuals. RNA was prepared using the RNeasy^TM^ mini kit from Qiagen and then DNased using the TURBO DNA-*free*™ Kit from Applied Biosystems. One sample of cirri at 20 PSU and one sample of cirri at 33 PSU contained very low RNA amounts and were therefore excluded in the following analyses. Thus, for cirri at 20 and 33 PSU only 5 replicates were used.

### cDNA synthesis and qPCR

For gene expression analyses, cDNA synthesis was performed on 200 ng DNAse treated RNA using the iScript™ cDNA Synthesis Kit (Bio-Rad). qPCR was performed utilizing primers for the different *B*. *improvisus* aquaporins (S6 Table) using in total 2 ng cDNA and SYBR Green supermix (Bio-Rad) for detection. Primers were designed to anneal to parts of the aquaporin sequences that had as high sequence identity as possible between individuals, but as high sequence difference as possible between aquaporin paralogs. The qPCR protocol was as follows: an initial denaturation temperature of 95°C for 3 min, a denaturation step at 95°C for 20 s, an annealing temperature of 58–60°C (see S6 Table) for 20 s and elongation at 72°C for 30 s. In total 40 PCR cycles were run. The aquaporin expression levels were normalized against actin levels, which were measured for each qPCR run. For average 2^-ct^ actin values at different salinities, see S11 Fig. There was no significant difference in actin expression in relation to salinity in any of the tissues (ANOVA; p < 0.05). Primer efficiencies were optimized by creating standard curves at different temperatures, and using different amounts of cDNA (obtained from a batch of about 300 cyprid larvae). Primer efficiencies in the final qPCR were in the range of 94–104%.

Statistical analysis of qPCR data obtained from adults exposed to different salinities (Fig 9), were analysed using linear mixed effects models in the program R using the *lmer* command (that fits a linear mixed-effects model to data via restricted (residual) maximum likelihood) in the *lme4* package [84, 85]. The tissue and salinity were treated as fixed factors, and replicate (pooled tissue from three adults—soma, cirri or mantle) treated as random. Orthogonal *post-hoc* contrasts of effects were tested among salinities and among tissues using Dunnett’s test in R using the *mcp* command (that computes contrast matrices for several multiple comparison procedures) in the package *multcomp* [86].

## Supporting information

S1 Fig Phylogenetic analysis including big brain and water aquaporins. A phylogenetic tree, including 264 water and big-brain related aquaporin sequences from *B*. *improvisus* and other arthropods, was constructed using MrBayes. 12 eGLPs from insects were also included, as well as 9 GLPs that were used as an outgroup. All accessions numbers are listed in S3 Table. The aquaporins are named mainly according to in which clade they appear in our analyses; however, hexapod water aquaporins (Drip or Prip) were named according to Stavang et al [22]. The full species name and the phylogenetic group is indicated for each sequence; Cr = Crustacea, Ch = Chelicerata, HI = Hexapoda-Insecta, HD = Hexapoda-Diplura and HC = Hexapoda-Collembola. In A, all single sequences are visible and in B branches are collapsed to aid in perceiving the relationship between the different clades/groups. The branch support values are MrBayes posterior probabilities in percent. Red arrows indicate the *B*. *improvisus* aquaporins. The scalebar shows substitutions per site.
(PDF)

S2 Fig Exon/intron structure of the *B*. *improvisus* aquaporins displayed to scale. The exons and introns of the *B*. *improvisus* aquaporins are shown to scale. Coding parts of exons are indicated in black, 5’ and 3’ UTR regions in grey and introns by thin lines. In several cases the whole intron was not obtained in the DNA sequencing data and the symbol "//" indicates that the minimum size of the intron is shown.
(PDF)

S3 Fig Conserved region in the big brain C-terminus. Alignment between the *B*. *improvisus* big brain (biBib) and the *Drosophila* big brain aquaporin (dmBib) shows a 26 amino acid region that is 70% conserved between the two species. The conserved region is marked with a box and transmembrane helices in the *B*.*improvisus* Bib, as predicted with TMHMM, are underlined. Stars indicated the seven conserved tyrosines between the two species.
(PDF)

S4 Fig TMHMM predictions of the *B*. *improvisus* aquaporins. Predictions of the localization of the transmembrane (TM) helices of the *B*. *improvisus* aquaporins are shown. The plots show the probabilities of inside (blue line), outside (pink line) and TM helix (red bars). A) All *B*. *improvisus* aquaporins. B) Comparison of TMHMM predictions for Aqp12 from *B*. *improvisus*, *D*. *pulex*, *L*. *salmonis* and *H*. *sapiens*.
(PDF)

S5 Fig Tyrosines protruding into the pores of the BIB and BIB-like proteins. The *B*. *improvisus* Bib, BibL1 and BibL2 aquaporins are viewed from the extracellular side. For Bib and BibL1 one tyrosine in each protein is protruding into the pore, whereas in the case of BibL2 there are two. The protruding tyrosines might partially block transport. A) Bib B) BibL1 C) BibL2.
(PDF)

S6 Fig Expression of *B*. *improvisus* aquaporins in different tissues of adults. The figure is based on the same data as Fig 9 and compares aquaporin expression between different tissues at indicated salinities. Asterisks indicate significant levels (ANOVA): *** p<0.001, ** p<0.01, * p<0.05. Error bars show standard deviation.
(PDF)

S7 Fig Superposition of the *B*. *improvisus* Aqp1_v1 and Aqp2_v1 with their respective splice variants Aqp1_v2 and Aqp2_v2. Homology models of Aqp1_v1 and Aqp2_v1 are superposed to their respective longer splice variants Aqp1_v2 and Aqp2_v2. The C-termini of the longer splice variants are predicted to be differently positioned compared to that of the shorter variants. The longer splice variants are shown in green and the shorter splice variants in red. The yellow circle indicates were the amino acid sequence begins to differ between the two splice variants. The grey circle indicates the region with the NPA loops.
(PDF)

S8 Fig Detection of Aqp1 protein during the purification procedure. A Western blot detecting the his-tagged Aqp1 in the different protein purification steps was performed using anti-his antibodies. The oligomeric forms (monomer, dimer, trimer) that are typical for the migration of aquaporins in denaturing gels are indicated with red arrows.
(PDF)

S9 Fig Comassie staining and Westernblot of Glp1 and Glp2 in fractions eluted from the Ni-column. Comassie staining (A) and Western blot (B) of Glp1 and Glp2 was performed on selected fractions eluted from the Ni-column. Elution of the his-tagged Glp1 and Glp2 proteins bound to the Ni-column was performed in 1 ml fractions using elution buffer containing step-wise increasing amounts of imidazole (2x40 mM, 4x80 mM, 5x250 mM and 5x400 mM). Not all fractions were analysed. Anti-his antibodies were used for the Western blot. Lane 1 is unbound protein in the supernatant after incubation with the nickel gel. Lane 2 is protein after washing the gel with a larger volume of elution buffer containing 40mM imidazole. Fractions pooled, concentrated and used for liposome reconstitution are indicated with a bracket (lane 9–17 for the comassie staining (A) and lane 6–14 for the western blot (B)). In B, the oligomeric forms (monomer, dimer, trimer) that are typical for the migration of aquaporins in denaturing gels are indicated with numbers. 1 = monomer, 2 = dimer, 3 = trimer.
(PDF)

S10 Fig Western blot of Aqp1, Glp1 and Glp2 in the reconstituted liposomes. Western blot was performed on reconstituted liposomes using an anti-his antibody. The results from two different experiments are shown. Control is a liposome without any added aquaporin protein. w = liposome from water transport analysis, g = liposome from glycerol transport analysis. The oligomeric forms (monomer, dimer, trimer) that are typical for the migration of aquaporins in denaturing gels are indicated with numbers. 1 = monomer, 2 = dimer, 3 = trimer.
(PDF)

S11 Fig mRNA expression of actin at different salinities. Adult individuals were incubated for 14 days at three different salinities (3, 20 and 33 PSU). For RNA preparation, soma, cirri and mantle were separated. For each salinity, the tissues (soma, cirri or mantle) from eighteen adults were pooled three-by-three to give six independent samples (n = 6). Quantitative PCR was used to determine actin expression levels to be used for normalization of AQP expression. In case of cirri at 20 and 33 PSU only 5 independent samples were used in the qPCR due to very low RNA amounts obtained from one of the samples in each case. Actin expression was measured six times for each of the 18 pooled samples in six different runs (one time for each of the aquaporins). Actin 2^-ct^ values were normalized against the average of the 2^-ct^ values of all 18 samples in the same run. An average of six the different runs are shown. Error bars show the standard deviation.
(PDF)

S1 Table ANOVA analysis of aquaporin expression in cyprids cultured at 33 PSU (For Fig 8B). (PDF)

S2 Table Primers for cloning of *B*.*improvisus* aquaporins. (PDF)

S3 Table Accession numbers used in S1 Fig. (XLSX)

S4 Table Parameters for the homology modeling. (PDF)

S5 Table Templates used in the homology modeling of aquaporins. Main template in bold; templates not used in final improved model in italics.
(PDF)

S6 Table Primers for qPCR. (PDF)

S1 AQP sequences Protein sequences of the *Balanus improvisus* aquaporins and their accession numbers. (DOCX)

S1 Model Homology 3D model for Aqp1_v1. (PDB)

S2 Model Homology 3D model for Aqp1_v2. (PDB)

S3 Model Homology 3D model for Aqp2_v1. (PDB)

S4 Model Homology 3D model for Aqp2_v2. (PDB)

S5 Model Homology 3D model for Glp1. (PDB)

S6 Model Homology 3D model for Glp2. (PDB)

S7 Model Homology 3D model for Aqp12. (PDB)

S8 Model Homology 3D model for Bib. (PDB)

S9 Model Homology 3D model for BibL1. (PDB)

S10 Model Homology 3D model for BibL2. (PDB)
