# Supplementary material for: Analysis of aquaporins from the euryhaline barnacle Balanus improvisus reveals differential expression in response to changes in salinity
Source: PLoS One. 2017 Jul 17;12(7):e0181192. doi: 10.1371/journal.pone.0181192 (PMC5513457; doi:10.1371/journal.pone.0181192)
Supplement: S1 Fig — A phylogenetic tree, including 264 water and big-brain related aquaporin sequences from B. improvisus and other arthropods, was constructed using MrBayes. 12 eGLPs from insects were also included, as well as 9 GLPs that were used as an outgroup. All accessions numbers are listed in S3 Table. The aquaporins are named mainly according to in which clade they appear in our analyses; however, hexapod water aquaporins (Drip or Prip) were named according to Stavang et al [22]. The full species name and the phylogenetic group is indicated for each sequence; Cr = Crustacea, Ch = Chelicerata, HI = Hexapoda-Insecta, HD = Hexapoda-Diplura and HC = Hexapoda-Collembola. In A, all single sequences are visible and in B branches are collapsed to aid in perceiving the relationship between the different clades/groups. The branch support values are MrBayes posterior probabilities in percent. Red arrows indicate the B. improvisus aquaporins. The scalebar shows substitutions per site. (PDF) [file pone.0181192.s001.pdf]

S1A Fig

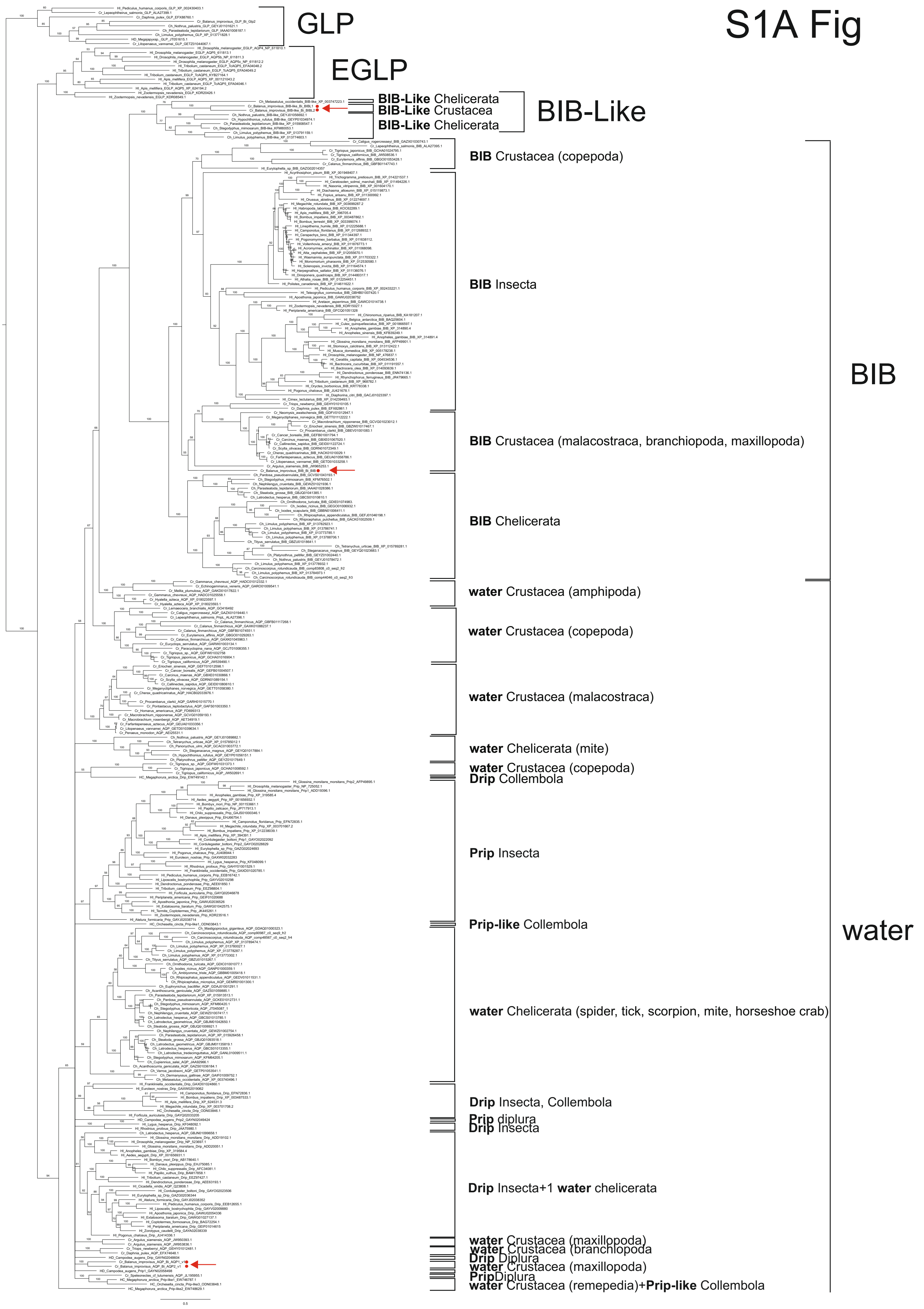
GLP
EGLP
BIB-Like Chelicerata
BIB-Like Crustacea
BIB-Like Chelicerata
BIB-Like
BIB Crustacea (copepoda)
BIB Insecta
BIB
BIB Crustacea (malacostraca, branchiopoda, maxillopoda)
BIB Chelicerata
water Crustacea (amphipoda)
water Crustacea (copepoda)
water Crustacea (malacostraca)
water Chelicerata (mite)
water Crustacea (copepoda)
Drip Collembola
Prip Insecta
Prip-like Collembola
water
water Chelicerata (spider, tick, scorpion, mite, horseshoe crab)
Drip Insecta, Collembola
Prip diplura
Drip Insecta
Drip Insecta+1 water chelicerata
water Crustacea (maxillopoda)
water Crustacea (branchiopoda
Drip Diplura
water Crustacea (maxillopoda)
Prip Diplura
water Crustacea (remepedia)+Prip-like Collembola
0.5

S1B Fig

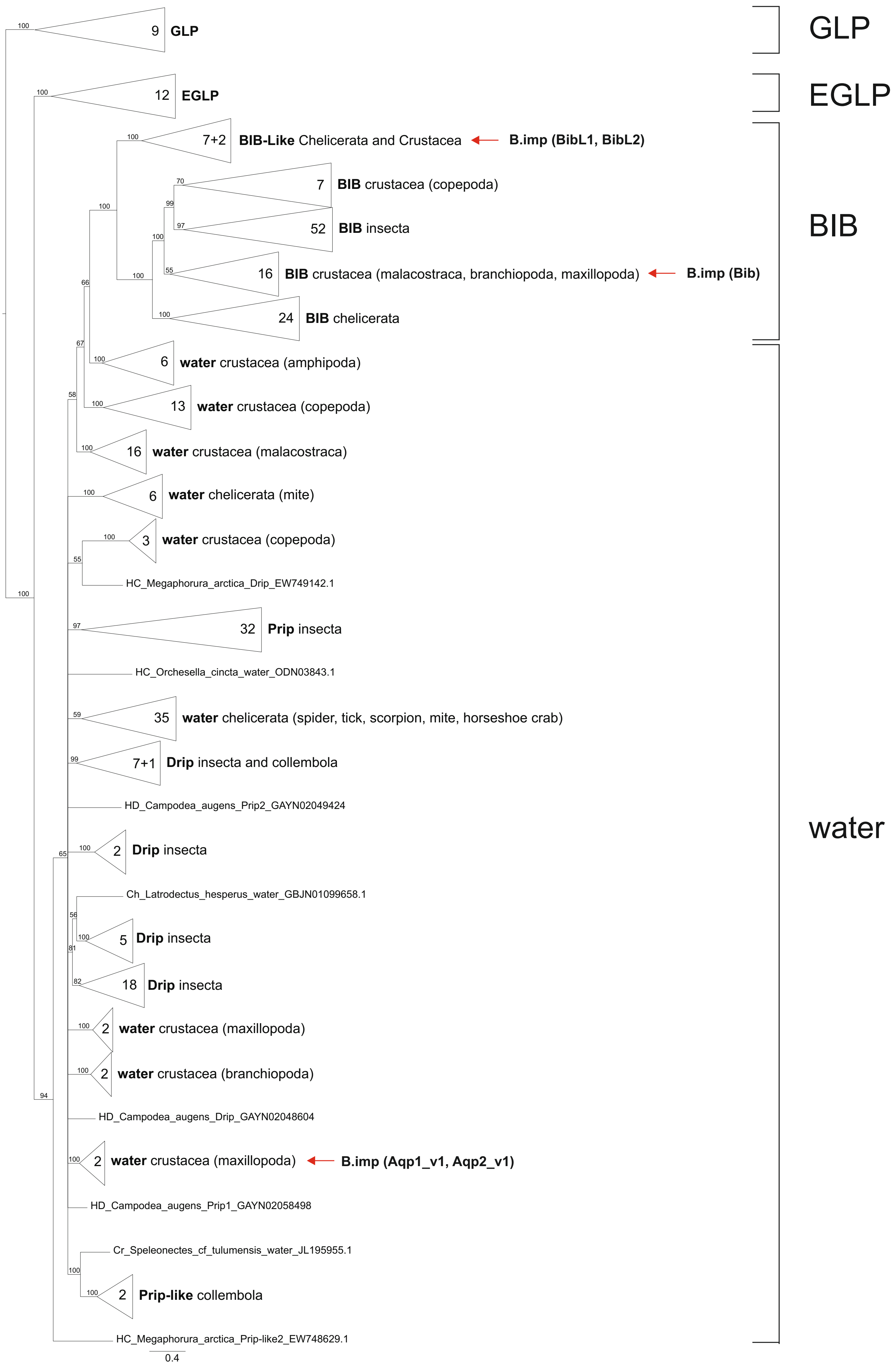
9 GLP
12 EGLP
7+2 BIB-Like Chelicerata and Crustacea
B.imp (BibL1, BibL2)
7 BIB crustacea (copepoda)
52 BIB insecta
16 BIB crustacea (malacostraca, branchiopoda, maxillopoda)
B.imp (Bib)
24 BIB chelicerata
6 water crustacea (amphipoda)
13 water crustacea (copepoda)
16 water crustacea (malacostraca)
6 water chelicerata (mite)
3 water crustacea (copepoda)
HC_Megaphorura_arctica_Drip_EW749142.1
32 Prip insecta
HC_Orchesella_cincta_water_ODN03843.1
35 water chelicerata (spider, tick, scorpion, mite, horseshoe crab)
7+1 Drip insecta and collembola
HD_Campodea_augens_Prip2_GAYN02049424
2 Drip insecta
Ch_Latrodectus_hesperus_water_GBJN01099658.1
5 Drip insecta
18 Drip insecta
2 water crustacea (maxillopoda)
2 water crustacea (branchiopoda)
HD_Campodea_augens_Drip_GAYN02048604
2 water crustacea (maxillopoda)
B.imp (Aqp1_v1, Aqp2_v1)
HD_Campodea_augens_Prip1_GAYN02058498
Cr_Speleonectes_cf_tulumensis_water_JL195955.1
2 Prip-like collembola
HC_Megaphorura_arctica_Prip-like2_EW748629.1
0.4
GLP
EGLP
BIB
water
