# Supplementary figures and images for: Analysis of aquaporins from the euryhaline barnacle Balanus improvisus reveals differential expression in response to changes in salinity
Source: PLoS One. 2017 Jul 17;12(7):e0181192. doi: 10.1371/journal.pone.0181192 (PMC5513457; doi:10.1371/journal.pone.0181192)

# S2 Fig

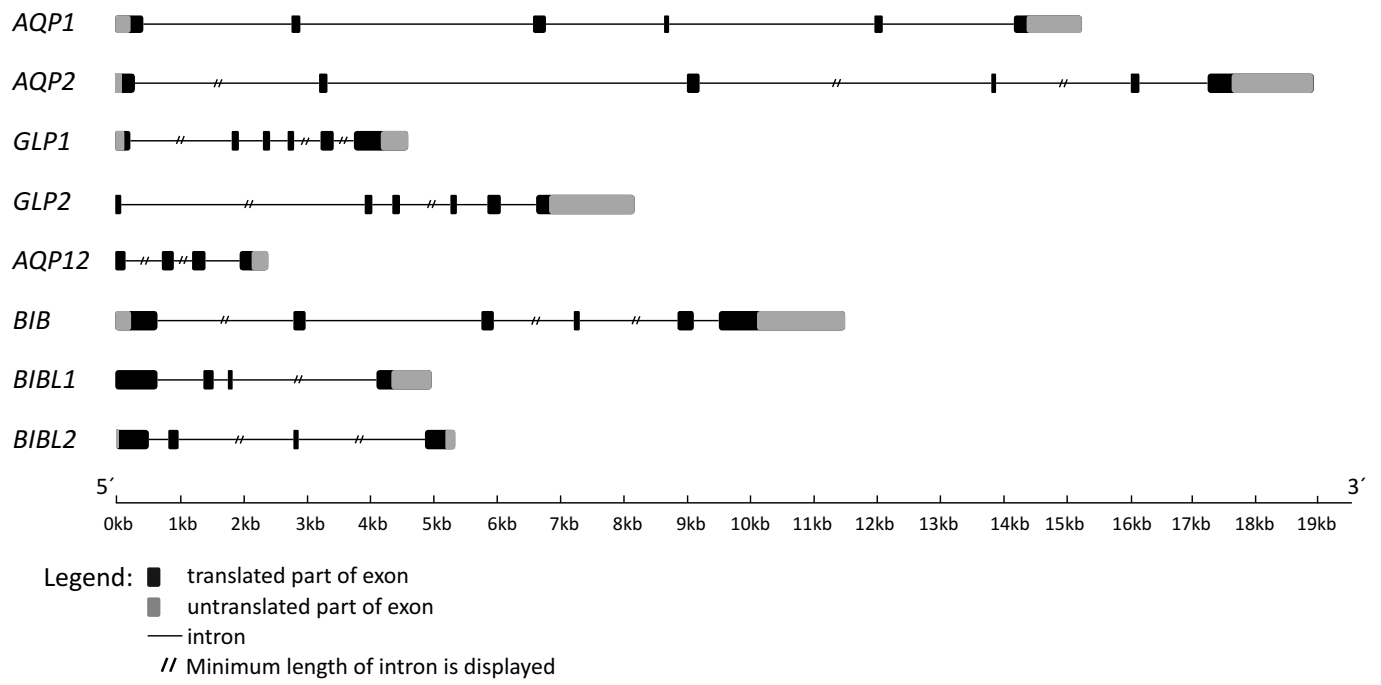

Supplement: S2 Fig — The exons and introns of the B. improvisus aquaporins are shown to scale. Coding parts of exons are indicated in black, 5’ and 3’ UTR regions in grey and introns by thin lines. In several cases the whole intron was not obtained in the DNA sequencing data and the symbol "//" indicates that the minimum size of the intron is shown. (PDF) [file pone.0181192.s002.pdf]

# S4A Fig

Aqp1\_v1

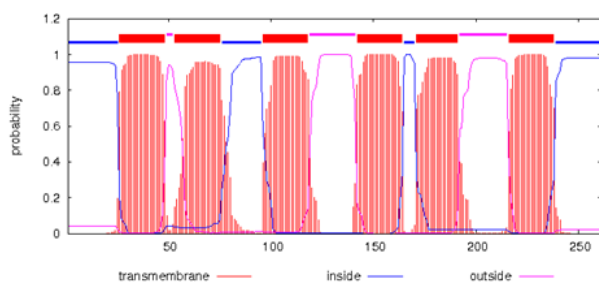

Bib

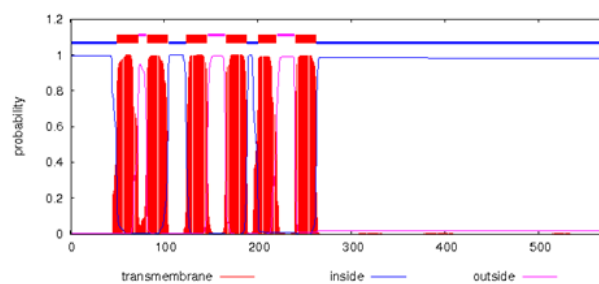

Aqp1\_v2

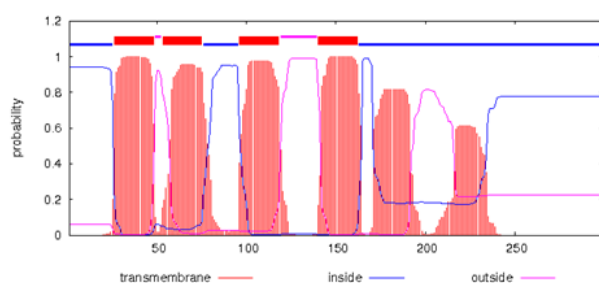

BibL1

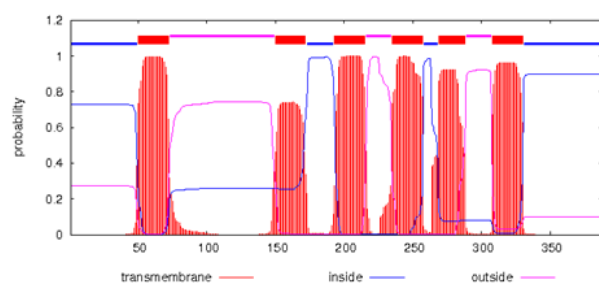

Aqp2\_v1

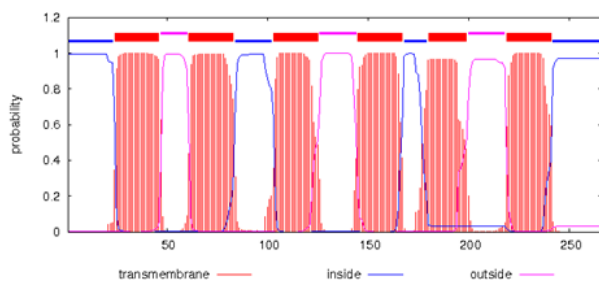

BibL2

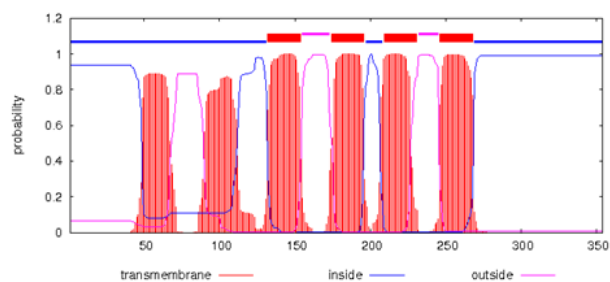

Aqp2\_v2

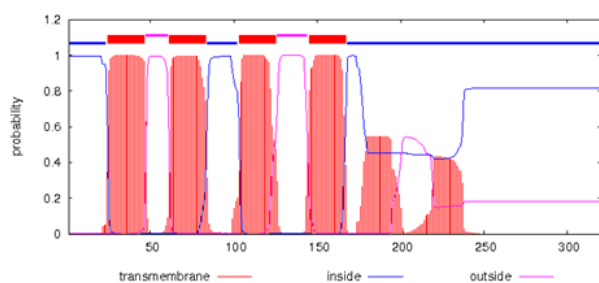

Glp1

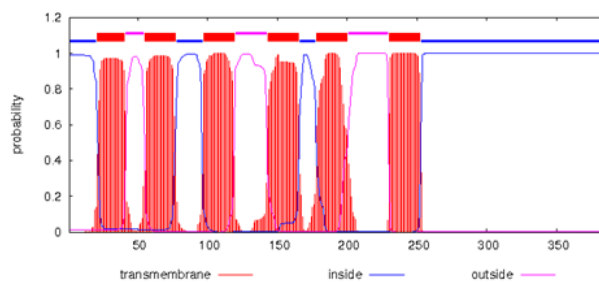

Aqp12

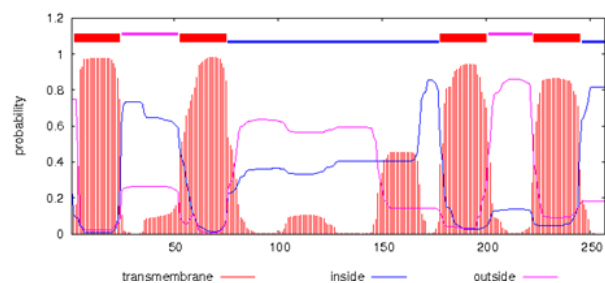

Glp2

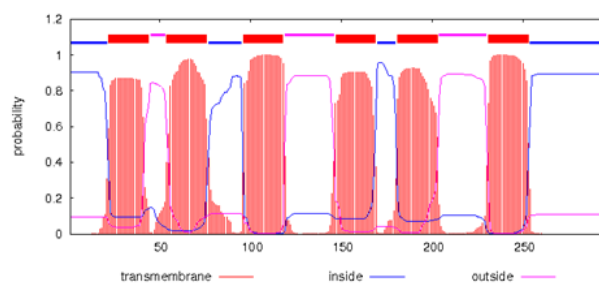

Bi\_Aqp12

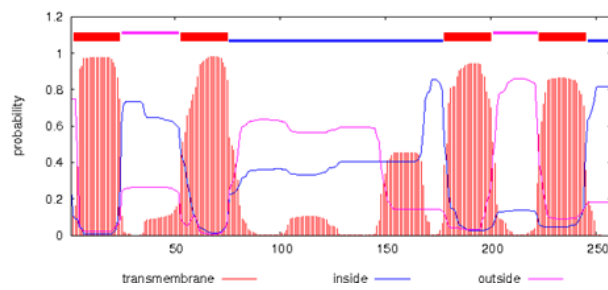

Dp\_Aqp12

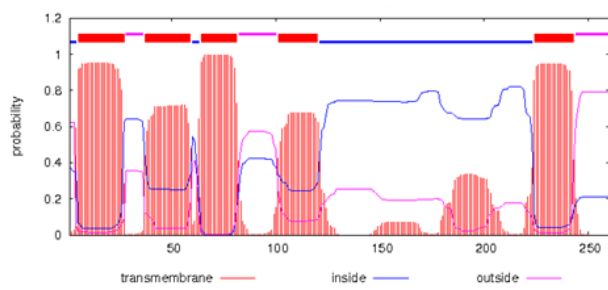

Ls\_Aqp12L1

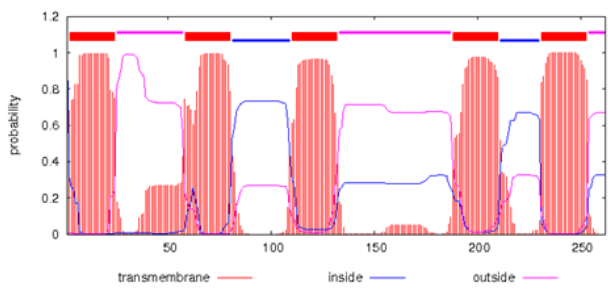

Ls\_Aqp12L2

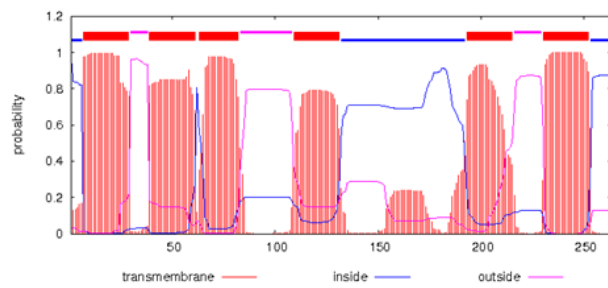

Hs\_AQP12

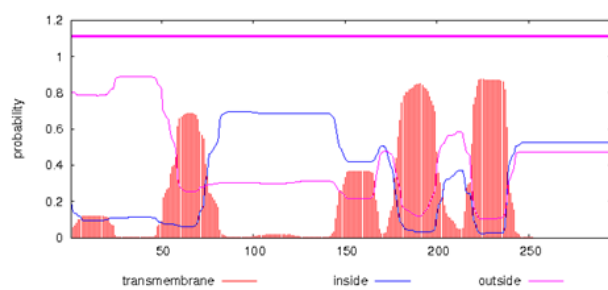

Supplement: S4 Fig — Predictions of the localization of the transmembrane (TM) helices of the B. improvisus aquaporins are shown. The plots show the probabilities of inside (blue line), outside (pink line) and TM helix (red bars). A) All B. improvisus aquaporins. B) Comparison of TMHMM predictions for Aqp12 from B. improvisus, D. pulex, L. salmonis and H. sapiens. (PDF) [file pone.0181192.s004.pdf]

A

Bib

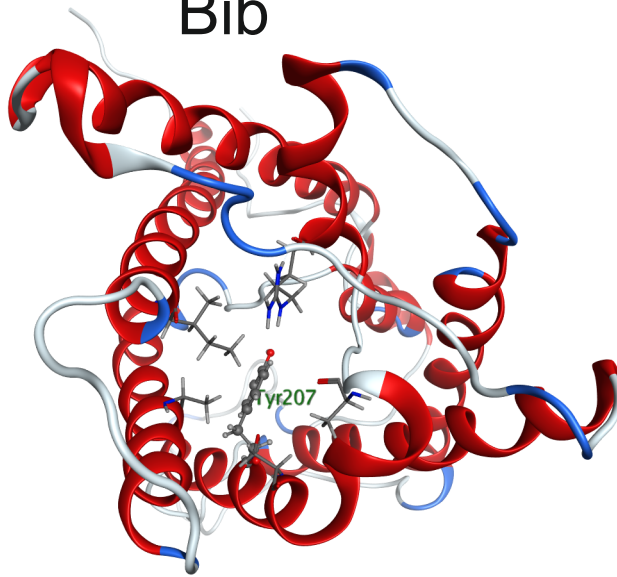

B

BibL1

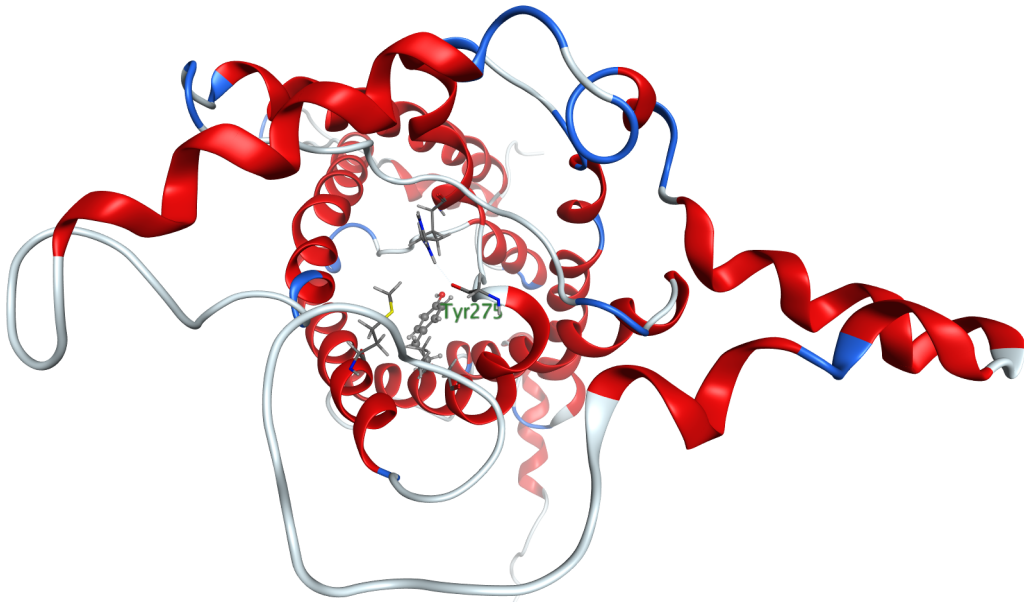

C

BibL2

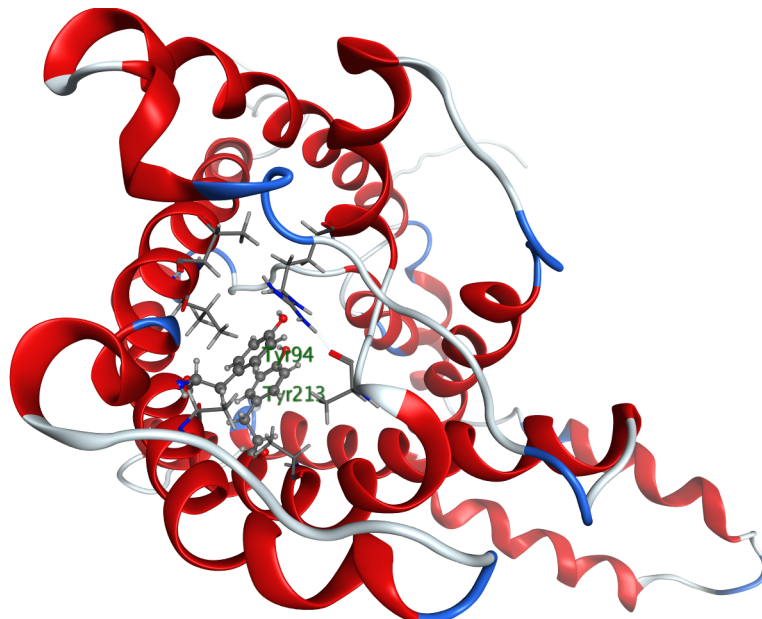

Supplement: S5 Fig — The B. improvisus Bib, BibL1 and BibL2 aquaporins are viewed from the extracellular side. For Bib and BibL1 one tyrosine in each protein is protruding into the pore, whereas in the case of BibL2 there are two. The protruding tyrosines might partially block transport. A) Bib B) BibL1 C) BibL2. (PDF) [file pone.0181192.s005.pdf]

# S6 Fig

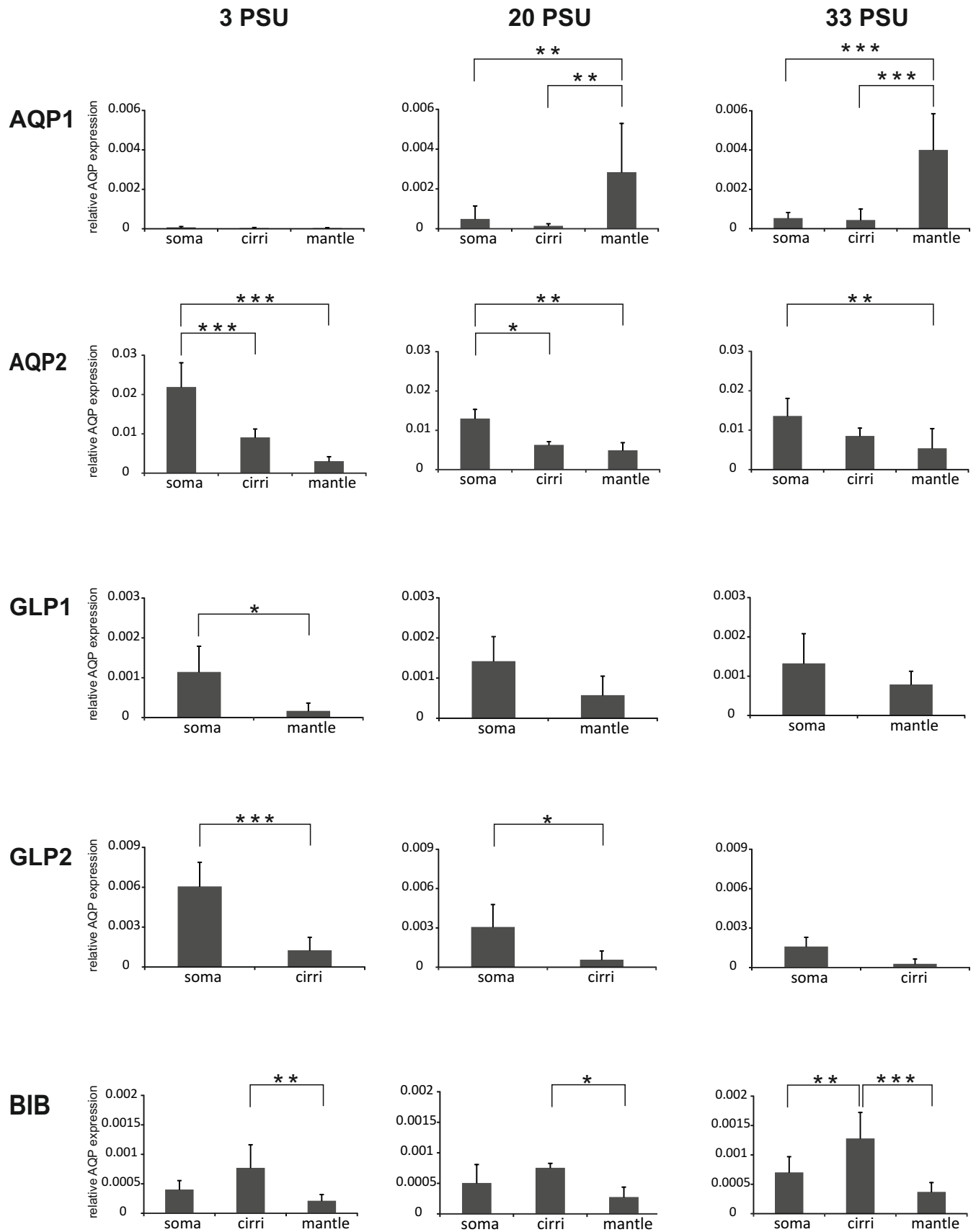

Supplement: S6 Fig — The figure is based on the same data as Fig 9 and compares aquaporin expression between different tissues at indicated salinities. Asterisks indicate significant levels (ANOVA): *** p<0.001, ** p<0.01, * p<0.05. Error bars show standard deviation. (PDF) [file pone.0181192.s006.pdf]

Aqp1

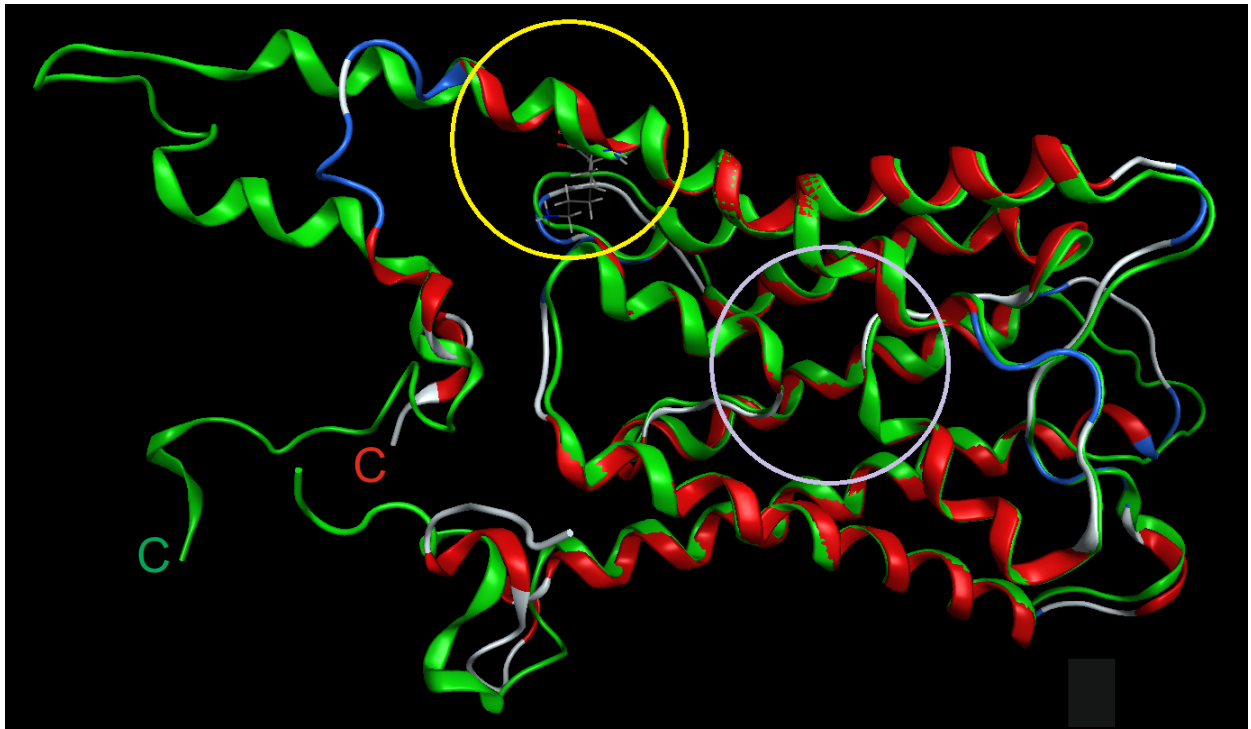

Aqp2

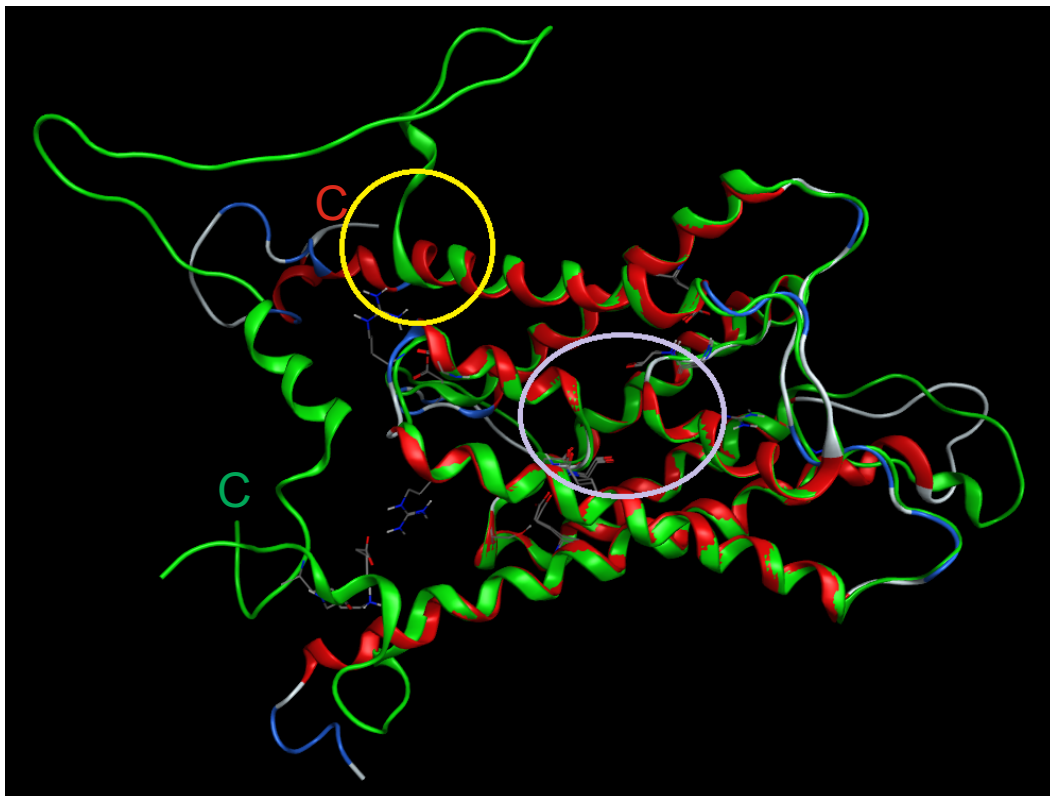

Supplement: S7 Fig — Homology models of Aqp1_v1 and Aqp2_v1 are superposed to their respective longer splice variants Aqp1_v2 and Aqp2_v2. The C-termini of the longer splice variants are predicted to be differently positioned compared to that of the shorter variants. The longer splice variants are shown in green and the shorter splice variants in red. The yellow circle indicates were the amino acid sequence begins to differ between the two splice variants. The grey circle indicates the region with the NPA loops. (PDF) [file pone.0181192.s007.pdf]

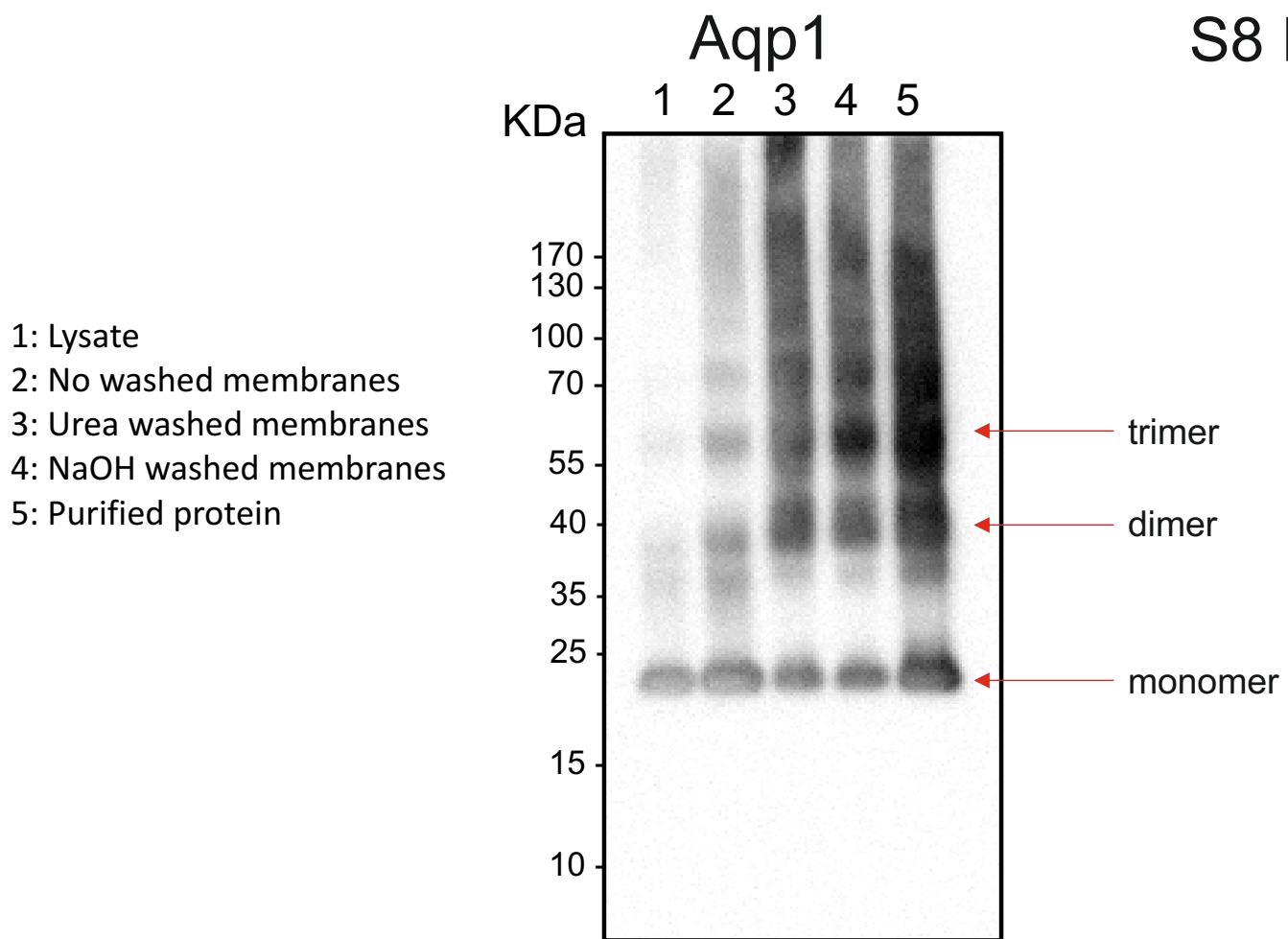

Supplement: S8 Fig — A Western blot detecting the his-tagged Aqp1 in the different protein purification steps was performed using anti-his antibodies. The oligomeric forms (monomer, dimer, trimer) that are typical for the migration of aquaporins in denaturing gels are indicated with red arrows. (PDF) [file pone.0181192.s008.pdf]

# S9 Fig

Glp1

Glp2

A)

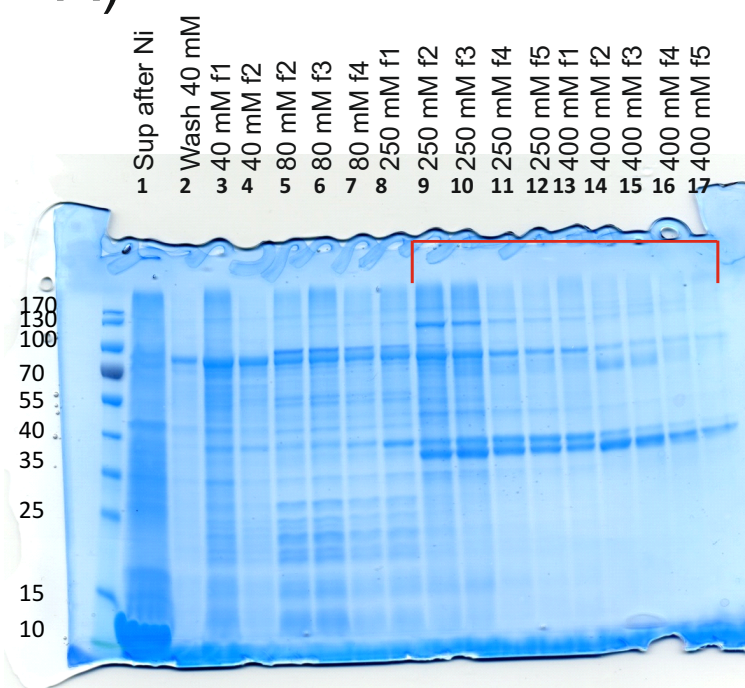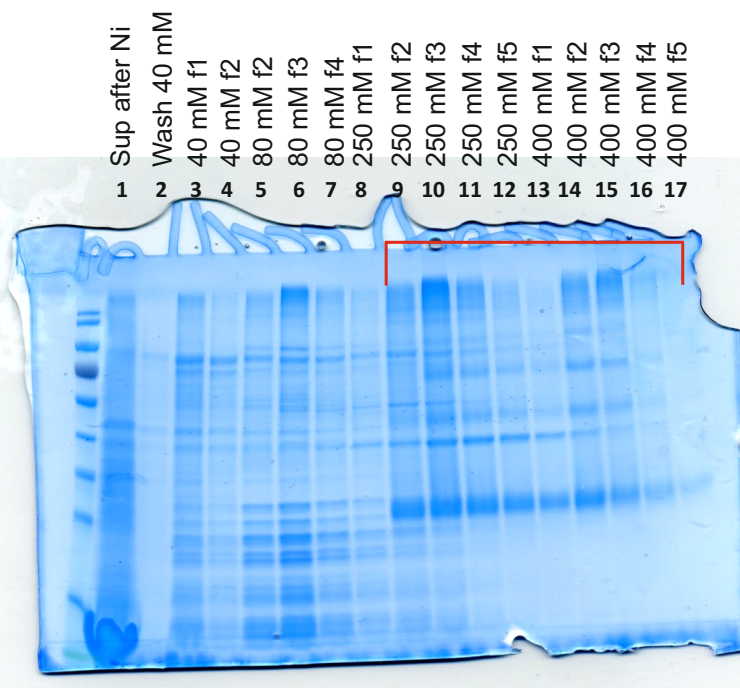

Glp1

Glp2

B)

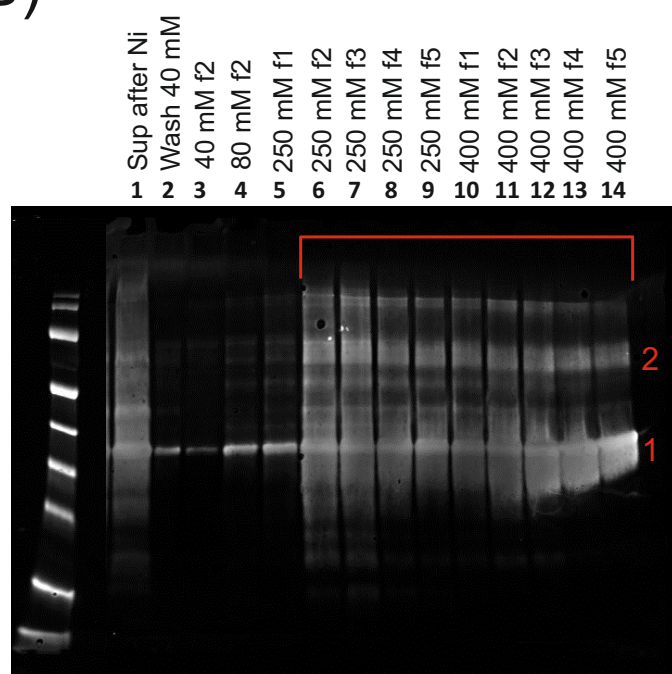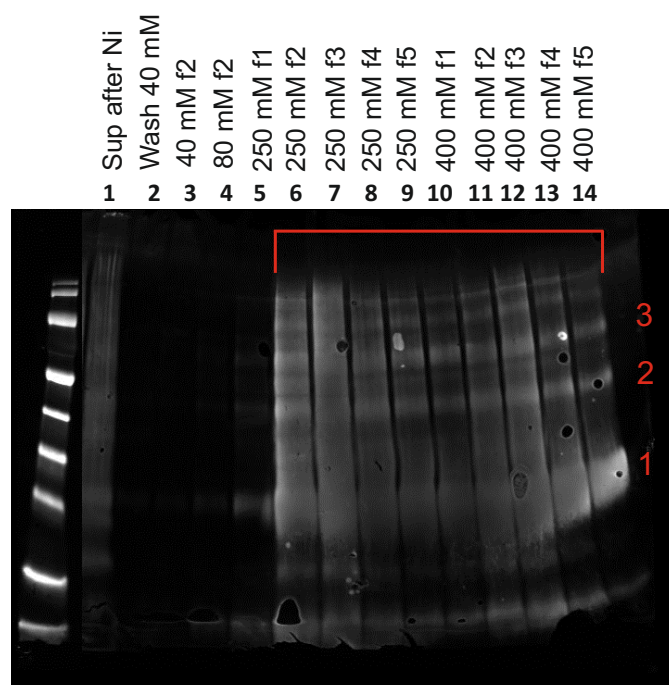

3=trimer  
2=dimer  
1=monomer

Supplement: S9 Fig — Comassie staining (A) and Western blot (B) of Glp1 and Glp2 was performed on selected fractions eluted from the Ni-column. Elution of the his-tagged Glp1 and Glp2 proteins bound to the Ni-column was performed in 1 ml fractions using elution buffer containing step-wise increasing amounts of imidazole (2x40 mM, 4x80 mM, 5x250 mM and 5x400 mM). Not all fractions were analysed. Anti-his antibodies were used for the Western blot. Lane 1 is unbound protein in the supernatant after incubation with the nickel gel. Lane 2 is protein after washing the gel with a larger volume of elution buffer containing 40mM imidazole. Fractions pooled, concentrated and used for liposome reconstitution are indicated with a bracket (lane 9–17 for the comassie staining (A) and lane 6–14 for the western blot (B)). In B, the oligomeric forms (monomer, dimer, trimer) that are typical for the migration of aquaporins in denaturing gels are indicated with numbers. 1 = monomer, 2 = dimer, 3 = trimer. (PDF) [file pone.0181192.s009.pdf]

S10 Fig

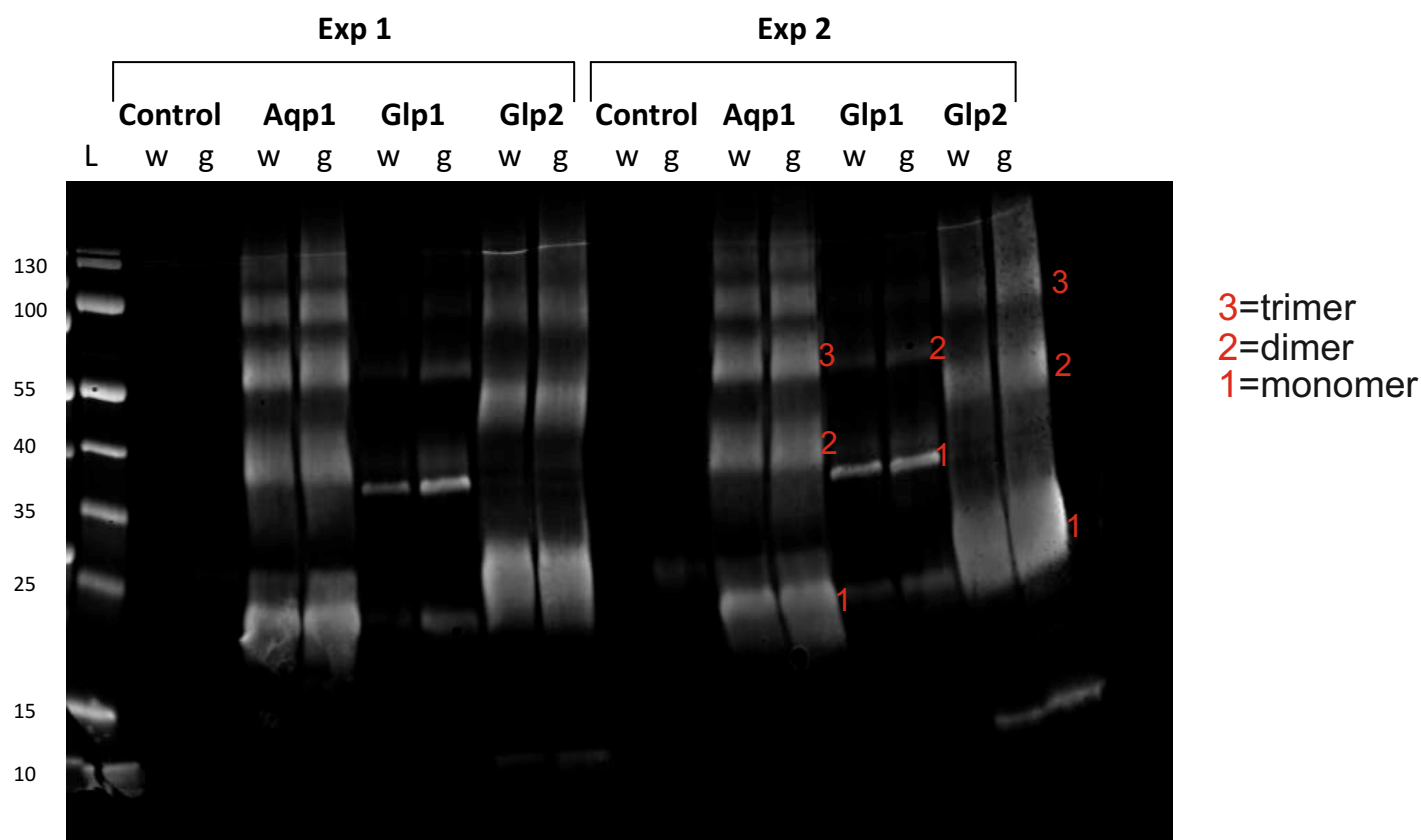

Supplement: S10 Fig — Western blot was performed on reconstituted liposomes using an anti-his antibody. The results from two different experiments are shown. Control is a liposome without any added aquaporin protein. w = liposome from water transport analysis, g = liposome from glycerol transport analysis. The oligomeric forms (monomer, dimer, trimer) that are typical for the migration of aquaporins in denaturing gels are indicated with numbers. 1 = monomer, 2 = dimer, 3 = trimer. (PDF) [file pone.0181192.s010.pdf]

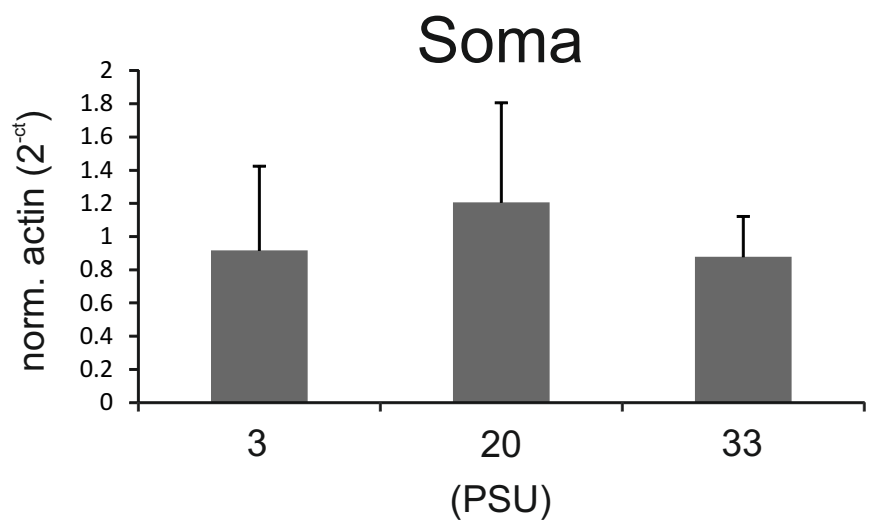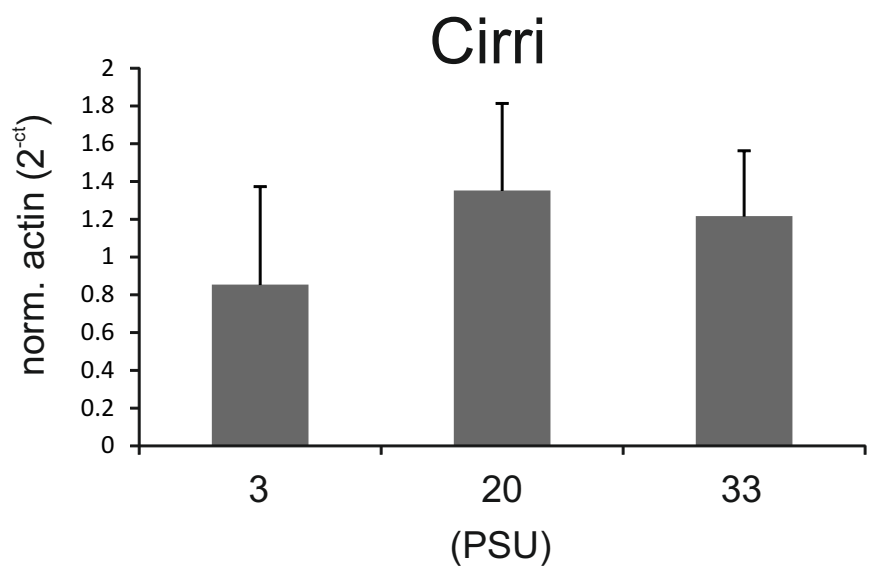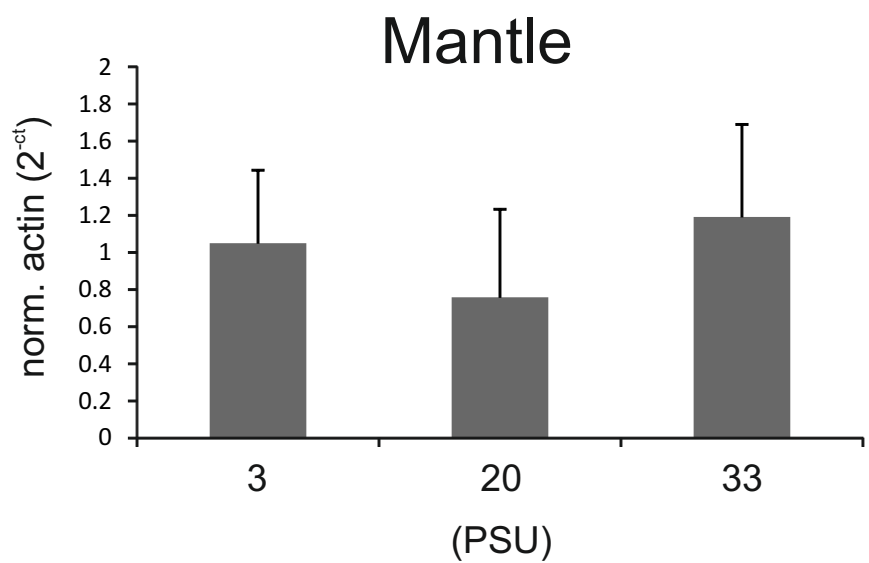

Supplement: S11 Fig — Adult individuals were incubated for 14 days at three different salinities (3, 20 and 33 PSU). For RNA preparation, soma, cirri and mantle were separated. For each salinity, the tissues (soma, cirri or mantle) from eighteen adults were pooled three-by-three to give six independent samples (n = 6). Quantitative PCR was used to determine actin expression levels to be used for normalization of AQP expression. In case of cirri at 20 and 33 PSU only 5 independent samples were used in the qPCR due to very low RNA amounts obtained from one of the samples in each case. Actin expression was measured six times for each of the 18 pooled samples in six different runs (one time for each of the aquaporins). Actin 2-ct values were normalized against the average of the 2-ct values of all 18 samples in the same run. An average of six the different runs are shown. Error bars show the standard deviation. (PDF) [file pone.0181192.s011.pdf]
