# Supplementary material for: Analysis of aquaporins from the euryhaline barnacle Balanus improvisus reveals differential expression in response to changes in salinity
Source: PLoS One. 2017 Jul 17;12(7):e0181192. doi: 10.1371/journal.pone.0181192 (PMC5513457; doi:10.1371/journal.pone.0181192)
Supplement: S3 Fig — Alignment between the B. improvisus big brain (biBib) and the Drosophila big brain aquaporin (dmBib) shows a 26 amino acid region that is 70% conserved between the two species. The conserved region is marked with a box and transmembrane helices in the B.improvisus Bib, as predicted with TMHMM, are underlined. Stars indicated the seven conserved tyrosines between the two species. (PDF) [file pone.0181192.s003.pdf]

S3 Fig

```
DmBib   MADESLHTVPLEHNIDYHIVTLFERLEAMRKDSHGGGHGVNNRLSSTLQAPKRSMQAEIRTLEFWRSIISECLASFMYVFIVCGAAAGVG
BiBib   MAITSL----SAETLDSHILALLDKLDHVQGEL-------------SPPPARLPMHVEVRRLEFWRAIIAECMATFFFVFLICAANVPWS

DmBib   VGASVSSVLLATALASGLAMATLTQCFLHISGAHINPAVTLALCVVRSISPIRAAMYITAQCGGGIAGAALLYGVTVPGYQGNLQAAISH
BiBib   THW-ASQSLIAGAFTAGFAAAALTQCFWRVSGAHMNPAVTLAHASTRKISPLRCLLYVTAQCGGAIAGAALLYGSSTTSLQGSLGVTVV-

DmBib   SAALAAWERFGVEFILTFLVVLCYFVSTDPMKKFMGNSAASIGCAYSACCFVSM----PYLNPARSLGPSFVLNKWDSHWVYWFGPLVGG
BiBib   TPPLTAWQGFGVEFVLTFILVFVVFSVSEPSRRPLGNSSVVIGFTYLAVSLAGIRCTGASMNPARSLGPAFVMNIWKDHWVYWFSPLTAA

DmBib   MASGLVYEYIFNSRNRNLRHNKGSIDNDSSSIHSEDELNYD-MDMEKPNKYQQSQ----GTYPRGQSNGNGGGQAAGNGQHQAANMGQMP
BiBib   VIAAYVHEYIFNPNRRYRL--KDTMDNESLGGQSDDEVFDEERPKLSPADYNTLRSQTYAAYAPASKASPAG------------------

                                        *      *                 *                        *
DmBib   GVVANAGQGNYCQNLYTAPPLSSKYDQQQEPLYGGTRSLYCRSPTLTRSNLNRSQSVYAKSNTAINRDIVPRPGPLVPAQSLYPMRTQQQ
BiBib   ----------GARSVFSVPAYR-GGVSRAESVYGGTKSLYACSPPPSRANLARSQSVYTKERRCG---MEPRAG-ITAAQSVYPRIGGGG

                                            *
DmBib   QQQQQQQQQQVAPAPQSSHLQNQNVQNQMQQRSESIYGMRGSM----------RGQQQPIQQQ--QQQQQQQQLQQQQPNMGVQQQQMQP
BiBib   GGLAESMYATRAPRQETVYRPAEPVDS----QKQQLYSAKEDALSYSTATTHFTASSRPDDYAAYSPRHDDYGVYGKQDLYGKQQDVYGK

                                                                     *  *
DmBib   PPQMMSDPQQQPQGFQPVYGTRTNPTPMDGNHKYDRRDPQQMYGVTGPRNRGQSAQSDDSSYGSYHGSAVTPPARHPSVEPSPPPPPML-
BiBib   QPELYAKPQ-YPGSYQSAA--------AENAHN--QRN-----ASGRPH---TSGGGGLPAYGAYSGRAGPPP----------PPPPRNG

DmBib   -MYAPPPQPNAAHPQPIRTQSERKVSAPVVVSQPAACAVTYTTSQGSAVTAQQQQQQQQQQQQQQQQQQQQMMMQQQQQHYGMLPLRPN
BiBib   DMMSPRSMAGSET--------TSGLTTPNSVSS-------YR*----------------------------------------------
```
