# Supplementary material for: Analysis of aquaporins from the euryhaline barnacle Balanus improvisus reveals differential expression in response to changes in salinity
Source: PLoS One. 2017 Jul 17;12(7):e0181192. doi: 10.1371/journal.pone.0181192 (PMC5513457; doi:10.1371/journal.pone.0181192)
Supplement: S1 Table — (PDF) [file pone.0181192.s012.pdf]

S1 Table. ANOVA analysis of aquaporin expression in cyprids cultured at 33 PSU (For Fig 8B).

Multiple Comparisons

Dependent Variable: logex

Tukey HSD

(I) Gene	(J) Gene	Mean Difference (I-J)	Std. Error	Sig.	95% Confidence Interval Lower Bound	Upper Bound
AQP1	AQP12	.6234*	.12003	.001	.2243	1.0225
	AQP2	-.5677*	.12003	.002	-.9668	-.1686
	BIB	1.0888*	.12003	.000	.6897	1.4879
	BIBL1	1.2087*	.12003	.000	.8097	1.6078
	BIBL2	1.5462*	.12003	.000	1.1471	1.9453
	GLP1	1.2695*	.12965	.000	.8384	1.7005
	GLP2	1.1896*	.12003	.000	.7905	1.5887
AQP12	AQP1	-.6234*	.12003	.001	-1.0225	-.2243
	AQP2	-1.1911*	.12003	.000	-1.5902	-.7920
	BIB	.4654*	.12003	.015	.0664	.8645
	BIBL1	.5854*	.12003	.001	.1863	.9844
	BIBL2	.9228*	.12003	.000	.5238	1.3219
	GLP1	.6461*	.12965	.001	.2150	1.0771
	GLP2	.5662*	.12003	.002	.1672	.9653
AQP2	AQP1	.5677*	.12003	.002	.1686	.9668
	AQP12	1.1911*	.12003	.000	.7920	1.5902
	BIB	1.6565*	.12003	.000	1.2574	2.0556
	BIBL1	1.7764*	.12003	.000	1.3774	2.1755
	BIBL2	2.1139*	.12003	.000	1.7148	2.5130
	GLP1	1.8372*	.12965	.000	1.4061	2.2682

	GLP2	1.7573*	.12003	.000	1.3582	2.1564
BIB	AQP1	-1.0888*	.12003	.000	-1.4879	-.6897
	AQP12	-.4654*	.12003	.015	-.8645	-.0664
	AQP2	-1.6565*	.12003	.000	-2.0556	-1.2574
	BIBL1	.1199	.12003	.970	-.2792	.5190
	BIBL2	.4574*	.12003	.017	.0583	.8565
	GLP1	.1806	.12965	.851	-.2504	.6117
	GLP2	.1008	.12003	.989	-.2983	.4999
BIBL1	AQP1	-1.2087*	.12003	.000	-1.6078	-.8097
	AQP12	-.5854*	.12003	.001	-.9844	-.1863
	AQP2	-1.7764*	.12003	.000	-2.1755	-1.3774
	BIB	-.1199	.12003	.970	-.5190	.2792
	BIBL2	.3375	.12003	.141	-.0616	.7366
	GLP1	.0607	.12965	1.000	-.3703	.4918
	GLP2	-.0191	.12003	1.000	-.4182	.3800
BIBL2	AQP1	-1.5462*	.12003	.000	-1.9453	-1.1471
	AQP12	-.9228*	.12003	.000	-1.3219	-.5238
	AQP2	-2.1139*	.12003	.000	-2.5130	-1.7148
	BIB	-.4574*	.12003	.017	-.8565	-.0583
	BIBL1	-.3375	.12003	.141	-.7366	.0616
	GLP1	-.2768	.12965	.424	-.7078	.1543
	GLP2	-.3566	.12003	.103	-.7557	.0425
GLP1	AQP1	-1.2695*	.12965	.000	-1.7005	-.8384
	AQP12	-.6461*	.12965	.001	-1.0771	-.2150
	AQP2	-1.8372*	.12965	.000	-2.2682	-1.4061
	BIB	-.1806	.12965	.851	-.6117	.2504
	BIBL1	-.0607	.12965	1.000	-.4918	.3703
	BIBL2	.2768	.12965	.424	-.1543	.7078

	GLP2	-.0799	.12965	.998	-.5109	.3512
GLP2	AQP1	-1.1896*	.12003	.000	-1.5887	-.7905
	AQP12	-.5662*	.12003	.002	-.9653	-.1672
	AQP2	-1.7573*	.12003	.000	-2.1564	-1.3582
	BIB	-.1008	.12003	.989	-.4999	.2983
	BIBL1	.0191	.12003	1.000	-.3800	.4182
	BIBL2	.3566	.12003	.103	-.0425	.7557
	GLP1	.0799	.12965	.998	-.3512	.5109

Based on observed means.

The error term is Mean Square(Error) = .029.

*. The mean difference is significant at the 0
