# Supplementary material for: Analysis of aquaporins from the euryhaline barnacle Balanus improvisus reveals differential expression in response to changes in salinity
Source: PLoS One. 2017 Jul 17;12(7):e0181192. doi: 10.1371/journal.pone.0181192 (PMC5513457; doi:10.1371/journal.pone.0181192)
Supplement: S2 Table — (PDF) [file pone.0181192.s013.pdf]

S2 Table. **Primers for cloning of *B. improvisus* aquaporins**

**AQP**	Fw primer	Rev primer	Anneal. temp.	polymerase
AQP1	CGCCCCGCTCCTGATCACGAC	CAGGAGCGGCCCGAGCCAGTA	60 °C	Pfu ultra
AQP2	CTCCCCGCCACTGCACCGTCA	GAACATCGGGCCCAGCCAG	60 °C	Pfu ultra
GLP1	TACCTGACCTGACGTGACCT	CATTCACACAACGTTGCCTTC	55 °C	Pfu ultra
GLP2	GCAGGTGGCAGATCGGAGA	CATTCAGCGAAGTGCCGAGT	53 °C	Pfu ultra
AQP12	GGGAACATCAACATCTGGACA	CCCTCAGTCTCTCTTGGCGT	55 °C	Pfu ultra
BIB	TACCGGTACCGTCTCTCGAG	GCACTAGCCGCACCCTGTC	55 °C	Exp. Hi. Fi.
BIBL1	ACCGGACCACTCGCTGACA	CATACATATTCACAGGTCACATC	55 °C	Pfu ultra
BIBL2	GCAGGGTGGCGTCGTAGTT	AGTACGACTGTGCGTGTTGCT	55 °C	Pfu ultra
	5´race primer touch down	5´race primer nested		
AQP2	GAATGCCCGCATCAGGCTGACCT	CCACCAGCATGCCCGCCGTCA	65 °C (td), 60 °C (nest)	Pfu ultra
BIBL1	CGAGAGTCGTCACCACGGCGATA	TGGCGTGAGTCCCAGCACGATGA	65 °C (td), 63 °C (nest)	Pfu ultra
	3´race primer	3´race primer nested		
AQP2	CGCTCAGAGT ATCGGCCACA TCA	GGTGACGGCGGGCATGCTGGT	65 °C (td), 60 °C (nest)	Pfu ultra
BIBL1	GGCCACGTTTATCGCCGTGGTGA	CCATCGGGCTCGCCTACGGACT	65 °C (td), 62 °C (nest)	Pfu ultra
BIBL2	CTCAGGCGTTCGGCGCTGAGCT	CTCACTGACCAGGTGGCCTCTGT	65 °C (td), 61 °C (nest)	Pfu ultra

Abbreviations used: Anneal. temp, annealing temperature; Exp. Hi. Fi., Expand High Fidelity; td, touch down; nest, nested PCR
