# Supplementary material for: Analysis of aquaporins from the euryhaline barnacle Balanus improvisus reveals differential expression in response to changes in salinity
Source: PLoS One. 2017 Jul 17;12(7):e0181192. doi: 10.1371/journal.pone.0181192 (PMC5513457; doi:10.1371/journal.pone.0181192)
Supplement: S4 Table — (PDF) [file pone.0181192.s015.pdf]

S4 Table: Parameters for the homology modeling

Modeling speed (slow = best)	Slow
Number of PSI-BLAST iterations in template search	6
Maximum allowed PSI-BLAST E-value to consider template	0.5
Maximum number of templates to be used	5
Maximum number of templates with the same sequence	1
Maximum oligomerization state	4 (tetrameric)
Maximum number of alignment variations per template	5
Maximum number of conformations tried per loop	50
Maximum number of residues added to the termini	10
