# Supplementary material for: Analysis of aquaporins from the euryhaline barnacle Balanus improvisus reveals differential expression in response to changes in salinity
Source: PLoS One. 2017 Jul 17;12(7):e0181192. doi: 10.1371/journal.pone.0181192 (PMC5513457; doi:10.1371/journal.pone.0181192)
Supplement: S5 Table — Main template in bold; templates not used in final improved model in italics. (PDF) [file pone.0181192.s016.pdf]

S5 Table: Templates used in the homology modeling of aquaporins. Main template in bold; templates not used in final improved model in italics.

System	Residues in final model	Sequence identity / similarity[a]	Templates				
Aqp1_v1 261 residues	1-261	51.9 / 67.3	**3GD8 Human aquaporin 4**	3D9S Human aquaporin 5	3CN5 Spinach aquaporin Sopip2;1, S115E, S246E	*4NEF Human aquaporin 2*	*3C32 Eye lens aquaporin 0*
Aqp1_v2 299 residues	1-293	51.9/67.8	**3GD8 Human aquaporin 4**	3D9S Human aquaporin 5	3C32 Eye lens aquaporin 0	*5C5X human Aqp 5, S156E mutant*	*1SOR Aqp0*
Aqp2_v1 266 residues	6-266	51.6 / 67.6	**3GD8 Human aquaporin 4**	4NEF Human aquaporin 2	3CN5 Spinach aquaporin Sopip2;1, S115E, S246E	4JC6 Spinach aquaporin Sopip2;1	2C32 Eye lens aquaporin 0
Aqp2_v2 320 residues	1-288	51.6 / 67.1	**3GD8 Human aquaporin 4**	2C32 Eye lens aquaporin 0	3D9S Human aquaporin 5	4NEF Human aquaporin 2	*3CN5 Spinach aquaporin Sopip2;1, S115E, S246E*
AQP12 257 residues	21-255	21.4 / 38.0	**2O9D Aquaporin Aqpz T183C**	3NKA Aquaporin Aqpz H174G, T183F	2F2B Integral membrane protein aquaporin Aqpm	2O9E Aquaporin Aqpz T183C, with Hg	2O9G Aquaporin Aqpz L170C, with Hg
Glp1 383 residues	7-267	35.4 / 59.3	**1FX8 *E. coli* glycerol facilitator (Glpf) with substrate**	3C02 Aqua-glyceroporin from *P. falciparum*	3NE2 *A. fulgidus* aquaporin	*1LDF Glpf W48F, F200T mutant*	*2F2B Integral membrane protein aquaporin Aqpm*
Glp2 294 residues	7-294	33.0 / 57.3	**1LDF Glpf W48F, F200T mutant**	3C02 Aqua-glyceroporin from *P. falciparum*	1FX8 *E. coli* glycerol facilitator (Glpf) with substrate	3GD8 Human aquaporin 4	2F2B Integral membrane protein aquaporin Aqpm
Bib 570 residues	34-314	44.2 / 62.0	**3GD8 Human aquaporin**	2C32 Eye lens aquaporin 0	4JC6 Spinach aquaporin	4NEF Human aquaporin 2	3CN5 Spinach aquaporin

			**4**		Sopip2;1		Sopip2;1, S115E, S246E
BibL1 388 residues	11-359	40.3 / 56.6	**4NEF Human aquaporin 2**	2C32 Eye lens aquaporin 0	3D9S Human aquaporin 5	*3GD8 Human aquaporin 4*	*4JC6 Spinach aquaporin Sopip2;1*
BibL2 354 residues	27-324	39.8 / 55.7	**3GD8 Human aquaporin 4**	2C32 Eye lens aquaporin 0	4NEF Human aquaporin 2	3CN5 Spinach aquaporin Sopip2;1, S115E, S246E	*4JC6 Spinach aquaporin Sopip2;1*

[a] In %, compared to main template.
