# Supplementary material for: Analysis of aquaporins from the euryhaline barnacle Balanus improvisus reveals differential expression in response to changes in salinity
Source: PLoS One. 2017 Jul 17;12(7):e0181192. doi: 10.1371/journal.pone.0181192 (PMC5513457; doi:10.1371/journal.pone.0181192)
Supplement: S6 Table — (PDF) [file pone.0181192.s017.pdf]

S6 Table. Primers for qPCR

**AQP**	**qPCR fw primer**	**qPCR rev primer**	**Anneal. temp (ÁC)**
AQP1	ATCGTCATGCAGTGTATGG	GACGTGCTCGGATCGGG	58
AQP2	GCCTGGTCCACTCTGGAG	TGAGCGTGAGCAGCATGGT	58
GLP1	AGAGCTCGGGCAGAGGAA	TATCCTAAGGACTCGAGCTA	60
GLP2	GAGCCTTCTCTGACCAGGT	CCACGCAGAAGAGGTAGAC	60
AQP12	CTCTGGTGGCTGGAGCTG	GCCGCCCGAGTAGTTGAA	58
BIB	GCCTGGCTGAGTCCATGT	GAGTACGCAGCGTAGTCG	58
BIBL1	GGACTGGAGCATGTTTGAAT	GTCGTGGACGAACTCGTAG	58
BIBL2	GAGTCTCGGCAGCGTATC	TGGAGTCAGCCCGTACAG	58
actin	CATCAAGATCAAGATCATCGC	ATCTGCTGGAAGGTGGAC	58-60

Abbreviations used: Anneal. temp, annealing temperature
