# Supplementary material for: Analysis of aquaporins from the euryhaline barnacle Balanus improvisus reveals differential expression in response to changes in salinity
Source: PLoS One. 2017 Jul 17;12(7):e0181192. doi: 10.1371/journal.pone.0181192 (PMC5513457; doi:10.1371/journal.pone.0181192)
Supplement: S1 AQP sequences — (DOCX) [file pone.0181192.s018.docx]

**S1 AQP Sequences**

Protein sequences of the *Balanus improvisus* aquaporins and their accession numbers

>AQP1_1^a^ [PCR clone-KY508284]

MPAWSTAREGLGLQEVVNNKDLWKTLLAEFVGTLFLVFIGCLTCIGWTDE

GYAPSVVQIALGFGITVATMAQSIGHISGCHINPAVTVAMIVTRKIPLFR

ALCYIVMQCMGAAAGSALLKALTPEDIHGTLGMTQLNPKLTAAQGFGVEA

LITFVLVLVVFGVCDERREDVLGSGPLAIGLSVTTCHVGAIKYTGASMNP

ARSFGPALVTGLWDNHWVYWAGPVFGGLLAGGLYATAFRAGRLHSASDSD

REDTKMEKYEP*

>AQP1_2 [assembled from pacbio read-KY508286 and aa 1-40 of AQP1_1]

MPAWSTAREGLGLQEVV

NNKDLWKTLLAEFVGTLFLVFIGCLTCIGWTDEGYAPSVVQIALGFGITVATMAQSIGHI

SGCHINPAVTVAMLVTRNIPLFRALCYIVMQCMGAAAGSALLKALTPEDIHGTLGMTQLN

PKLTAAQGFGVEALITFVLVLVVFGVCDERREDVLGSGPLAIGLSVTTCHVGAIKYTGAS

MNPARSFGPALVTGLWENHWVYWLGPLLGGVLAGKLYKDCFLSEKLGTQKHHHHRTPRCA

DCGLLLPPHGGGEEEELKTRRSSRDPLVTGPEEGTGDKQELV*

>AQP2_1^b^ [PCR clone-KY508287]

MAWSTLEEALGLKEMVRNRDLWKALLAEFLGT

MLLTLIGCFSTIGWAANDDKDPYTPDMTQIALAFGITVATLAQSIGHISG

CHINPAVTAGMLVARQVSLMRAFFYIIVQCIGAIVGSALLKALTPEDVQG

SLGMTTVNPKLTAFQGFGVELFITFVLVFVVFGVCDEGRDDVKGSAPLAI

GLSITGCHLGAIKYTGSSMNPARTFGPAVVTGIWDNHWVYWAGPLAGGII

AGLVYTHAFRARHMAVAAVELTGLTEVKPQRGSK*

>AQP2_2 [Trinity contig-KY508289]

MAWSTLEEALGLKEMVRNRDLWKA

LLAEFLGTMLLTLIGCFSTIGWAANDDKDPYTPDMTQIALAFGITVATLAQSIGHISGCH

INPAVTAGMLVARQVSLMRAFFYIIVQCIGAIVGSALLKALTPEDVQGSLGMTTVNPKLT

AFQGFGVELFITFVLVFVVFGVCDEGRDDVKGSAPLAIGLSITGCHLGAIKYTGSSMNPA

RTFGPAVVTGIWDNHWVYWLGPMFGGCLAAKIYKDSFLACKLAPPLHCSVRRIVRGPEEP

EYETPAPEKKMLLDDVDGKMDGKMDGKMDGKLDGKMDGNGKPDDSTLSDPERTTDI*

>BIB [PCR clone-KY508290]

MAITSLSAETLDSHILALLDKLDHV

QGELSPPPARLPMHVEVRRLEFWRAIIAECMATFFFVFLICAANVPWSTHWASQSLIAGA

FTAGFAAAALTQCFWRVSGAHMNPAVTLAHASTRKISPLRCLLYVTAQCGGAIAGAALLY

GSSTTSLQGSLGVTVVTPPLTAWQGFGVEFVLTFILVFVVFSVSEPSRRPLGNSSVVIGF

TYLAVSLAGIRCTGASMNPARSLGPAFVMNIWKDHWVYWFSPLTAAVIAAYVHEYIFNPN

RRYRLKDTMDNESLGGQSDDEVFDEERPKLSPADYNTLRSQTYAAYAPASKASPAGGARS

VFSVPAYRGGVSRAESVYGGTKSLYACSPPPSRANLARSQSVYTKERRCGMEPRAGITAA

QSVYPRIGGGGGGLAESMYATRAPRQETVYRPAEPVDSQKQQLYSAKEDALSYSTATTHF

TASSRPDDYAAYSPRHDDYGVYGKQDLYGKQQDVYGKQPELYAKPQYPGSYQSAAAENAH

NQRNASGRPHTSGGGGLPAYGAYSGRAGPPPPPPPRNGDMMSPRSMAGSETTSGLTTPNS

VSSYR*

>BIBL1^c^ [assembled from PCR clone-KY508291 and DNA contig-KY508298]

MAGGLDGSSE

TALTPVELMLRYLESLPQEFSFREEVRSPKFWKALRAEFLASLLLVVFLSSGGIGIGVGV

GVGTGVGTGVGSGGGVGTHSGVGADVHAAATTISSSSSGGSNSGSGSSGHANPGPVISPS

AHSSNSSTSSGPLADTGAAVRMALAYCLTAATLIQCFGNVSGAQTNPAVTLSLLVTRYISPLRAAA

YVTVQLAGALCGALIVLGLTPPDWPDQPQVLAVASGVTLTQAFGVEFLAT

FIAVVTTLAVLDPVRADVGCKALSIGLAYGLGVLFSFQRTGACLNPVRAL

GPALVNNVWKDHWVYWVGPLFGGLLGGFTYEFVHDSSPHGRFLHRSFRRR

RGGQQPSRSGGALGRDVSGLSAATSELLQHPGTEDISTVSRH*

>BIBL2 [PCR clone-KY508294]

MAGGSDGREELAMSPVEMMRRYLDSLPHDFSVREELGSPKFWKAV

RAELLGSLLVAVFVTGAAAGHPRASGDGAGGDGGGDGGSDGGARAALAYA

LTAATLVQSLGSVSGAHANPAVTLCLLVRRLVSPLRAAVYVTAQLAGGLA

GALILYGLTPPGAALTDQVASVRPGVSLPQAFGAELLATFLVALATLAVL

EPVREDVGTKALSVGLAYGAGALFAFQLTGAGLNPARTLGPAVMYNVWTN

HWVYWVGPLFGGLLGGFTYEFVHEASAQGRLVRRSFRRRPCSSSDNGSSN

DGDDGGSGVRGQRDGLVPGQLGREVSGLSTTATDCLQLSTTTDCLQLSTT

EDASAAGRH*

>GLP1 [PCR clone-KY508295]

MILTMNRVRRACGIKHPIVRESLAELIGTATLVFFGDAAIANNMGSN

VGSTVNVPLGYATALALAVYVSGGVSGGHVNPCVTMGMCLTGRCDLNRLL

PFTIAQFIGAFLGAALTHGLFFDVFQLDLARSKSMYGVFATYPNPIISTM

GAFLDQTMGTAMLLFGIMAVTDAKNMNVPKGGIPACIGLVLFGVITASGS

NTGAALNPARDFSPRLYSYIIGYDNVFTEDDNFFWIPIVACYVGCVIGAF

LYFFCIEVHHPAELKDGEEQDVTTRIYKNYTTVVEPPTSRGPAGPAGRPA

GPSGRPAGPAGPAGPAGRPDSAPELGQRNGGFRDDADRPRSGGYPRDPGY

PKDSSYPRDPSYPRDPSYPRGPAYPRNESFRDERYM*

>GLP2 [PCR clone-KY508296]

MLLRLDRLRRACRLRHPLLRETLAELLGTTTLVFIGNS

VIANNVKNPLVASVNVPLGYVSAVIISVFATGGVSGGHVNPAVTLALCLA

GRCDLNRLLPFWGAQYLGGFLGALLTHILFFDLIQTDPDRINPAMYGIFA

TYPNDNISIGGAFSDQVLGTAILVFGCMAISDKRNMNVPKPMLPVAVGIV

LYGAISSSGSNTGAALNPARDFGPRIYSYMIGYQQVFSAGHCFWWIPVVA

CYIGGVLGAYVYLFCVELHHPVEDGQETMTITSAATPVDRKKTAWENAAM

ELEGNL*

>AQP12 [PCR clone-KY508297]

MSLAVSTSLILLACLLAHLLRLVVRRTVTADEPRRL

LHEAITASELCACAFELGIIANSYGVATYAVYLFLLIIYQSYAWGEVYAC

PYLNVEDWWLGRQPASRALLRILAEVAGGWLTWRYVRALWWLELAAGHRG

RWLETCTADLQVSPWHGALVEGVAVLLCRLVARALDERQHRFAAAIDSFC

ATTLVIAGFNYSGGYYNPMLASALKLGCHGHTVTEHLAVYWGAATAGALL

STQLYPLLRPALIARPDAKRD*

^a^ For AQP1_1 an additional sequence (KY508285), covering the ORF as well as parts of the 5´ and 3´ UTR, was obtained from a Trinity contig.

^b^ For AQP2_1 an additional sequence (KY5082889), covering the ORF as well as parts of the 5´ and 3´ UTR, was obtained from a Trinity contig.

^c^ For BIBL1, the sequences of two RNA contigs covering parts of the DNA contig- KY508298 has accession nr:s KY508292-93.
